# Macrophage membrane-biomimetic cinnamaldehyde nanomedicine ameliorates inflammatory bowel disease by suppressing macrophage M1 polarization

**DOI:** 10.7150/thno.124748

**Published:** 2026-03-25

**Authors:** Zebin Huang, Lingna Xie, Qi Shu, Yongyu Xu, Xiao-Chun Guo, Shimin Wang, Shuyi Li, Yaoxun Zeng, Xiu-Cai Chen, Mingtao Huang, Fujun Jin, Yu-Jing Lu

**Affiliations:** 1 School of Biomedical and Pharmaceutical Sciences, Guangdong University of Technology, Guangzhou, China.; 2 School of Food Science and Engineering, South China University of Technology, Guangzhou, China.

**Keywords:** cinnamaldehyde, macrophage polarization, mitophagy, IBD, macrophage membrane-biomimetic nanoplatform

## Abstract

**Rationale:**

Inflammatory bowel disease (IBD), known for its complexity and frequent relapses, urgently demands novel therapeutics due to the limited efficacy of current treatments. Cinnamaldehyde (CMA), a bioactive compound derived from *Cinnamomum cassia* Presl, has exhibited therapeutic potential for IBD. However, the therapeutic mechanism of CMA remains incompletely elucidated, and clinical translation is hampered by its poor oral pharmacokinetics.

**Methods:**

Using RAW 264.7 cells stimulated with either LPS or IL-4, we evaluated the effects of CMA on macrophage polarization. Subsequently, the impact of CMA on glucose metabolism in M1 macrophages was analyzed. RNA sequencing identified the signaling pathways through which CMA inhibits M1 macrophage polarization, and this was further validated through genetic or pharmacological blockade. To overcome the pharmacokinetic challenges of CMA, macrophage membrane-biomimetic CMA-loaded nanoparticles (MM@CMANP) were designed, and their pharmacokinetics and targeting to intestinal inflammation sites were evaluated. Finally, the efficacy of MM@CMANP was assessed in DSS-induced IBD mice.

**Results:**

CMA suppresses M1 macrophage polarization *in vitro*. Notably, CMA disrupted M1 macrophage glucose metabolic reprogramming, characterized by glycolysis suppression and enhanced oxidative phosphorylation. RNA sequencing demonstrated a clear association with mitophagy pathway following CMA treatment, and mechanistic studies verified that CMA promotes BCL2/adenovirus E1B 19 kDa-interacting protein 3 (BNIP3)-mediated mitophagy activation. Crucially, CMA-induced inhibition of M1 macrophages was mitigated by BNIP3 knockdown or autophagy inhibitors. MM@CMANP enhanced CMA accumulation in inflamed colonic tissues. In IBD mice, MM@CMANP significantly alleviated epithelial barrier disruption and mucosal inflammation. Consistent with *in vitro* findings, CMA modulated macrophage polarization and autophagy *in vivo*.

**Conclusions:**

These results establish mitophagy as a central mechanism underlying anti-IBD effects of CMA and position MM@CMANP as a clinically translatable nanotherapeutic platform for IBD.

## Introduction

Inflammatory bowel disease (IBD) represents a persistent, immune-driven inflammatory state of the gut, typically characterized by diarrhea with blood, abdominal cramps, and loss of body mass 1. By 2020, the global incidence of IBD had risen to approximately 6.8 million patients 2. Intestinal mucosal inflammation is the hallmark pathological feature of IBD, and accumulating evidence implicates macrophage dysfunction as a central driver of intestinal barrier disruption 3, 4. In IBD patients, disrupted M1/M2 polarization homeostasis drives over release of pro-inflammatory mediators, thereby exacerbating Th1/Th17-driven immune responses and compromising epithelial barrier integrity 5-7. Therefore, the treatment strategies for IBD primarily focuses on suppressing the inflammatory response. However, the therapeutic efficacy of current drugs remains suboptimal, particularly for antibody-based therapies that exhibit non-responsiveness or resistance in subsets of patients 8. Moreover, immunosuppressive therapies may increase the risks of infections and tumorigenesis 9. These limitations underscore the necessity to pursue safer, more efficacious interventions for IBD.

Natural products are increasingly viewed as attractive sources of new therapeutics, given their pleiotropic pharmacological actions and generally favorable safety profiles 10. *Cinnamomum cassia* Presl, a traditional Chinese herb with a long history in treating gastrointestinal disorders, has demonstrated efficacy in modern clinical and preclinical studies for alleviating diarrhea and IBD symptoms 11-13. China is the world's largest producer of *Cinnamomum cassia* Presl, with Guangdong Province accounting for over 40% of the nation's production. Cinnamaldehyde (CMA), the predominant constituent (more than 80%) of *Cinnamomum cassia* Presl essential oil, shows pleiotropic pharmacological effects, including antimicrobial, anti-inflammatory, anticancer potential, antidiabetic, and cardioprotective effects 14, 15. Several researches have revealed the potential of CMA in alleviating IBD symptoms in mice, likely attributable to its anti-inflammatory and immunomodulatory properties 16, 17. However, the mechanism by which CMA improves IBD pathology is not yet fully understood. Furthermore, the clinical translation of CMA is limited by a triad of physicochemical factors: its poor solubility in water, its inherent instability, and its insufficient bioavailability 18. Therefore, advanced delivery systems are necessary to enhance CMA's therapeutic performance.

Recently, the emergence of cell membrane biomimetic nanotechnology has provided new avenues for drug delivery, particularly for targeting tumors and central nervous system 19, 20. This innovative approach involves engineering nanoparticles (NP) coated with natural cell membranes, which endow these carriers with unique biofunctional properties such as immune escape, extended half-life in the bloodstream, and inherent tropism for specific tissues 21, 22. Noteworthily, macrophage membrane (MM)-derived coatings have become an attractive approach due to their potential to home to inflammatory sites and interact with microenvironmental cytokines 23. Such biomimetic nanoparticles have proven effective in enhancing targeted delivery and therapeutic outcomes of bioactive compounds in inflammatory disease models 24-26. Thus, this technology represents a viable means of surmounting the obstacles posed by CMA in IBD treatment.

Here, BNIP3-mediated mitophagy was identified as a key mechanism by which CMA suppresses M1 polarization, based on our investigation into its effects on macrophage phenotypes. Elucidating this mechanism provides key insights into how CMA inhibits inflammatory responses at the molecular level. Considering the limitations of CMA in terms of oral pharmacokinetics, we designed macrophage membrane-biomimetic CMA-loaded nanoparticles (MM@CMANP) and administered them intravenously, thereby bypassing first-pass metabolism associated with oral delivery. This biomimetic delivery system significantly enhanced targeting efficiency to inflamed colonic tissues and therapeutic efficacy of CMA, offering a novel and potent strategy for IBD.

## Methods

### Cell culture

RAW 264.7 macrophages were procured from the Wuhan Pricella Biotechnology Co., Ltd. (Procell, Cat. CL-0190). The cells were grown in Dulbecco's Modified Eagle Medium containing 10 % fetal bovine serum (FBS, ExCell Bio, Cat. FSP500), 100 U/L penicillin and 100 mg/L streptomycin. Cells were maintained at 37°C under 5% CO_2_ with humidity control.

### Macrophage polarization and CMA treatment

RAW 264.7 cells were driven toward either an M1 or M2 phenotype states using a slightly modified previous reported methods 27. Specifically, RAW 264.7 macrophages were seeded (5×10^5^ cells/mL) and cultured for 24 hours. Subsequently, after 1-h pretreatment with CMA (20 μM) or control medium, RAW 264.7 macrophages were exposed to lipopolysaccharide (LPS, Merck, Cat. L2630) at 1 μg/mL for M1 polarization, or with 20 ng/mL of interleukin-4 (IL-4, Novoprotein, Shanghai, China; Cat. CK15) for M2 polarization. After 24 h, the effects of CMA on RAW 264.7 macrophages were examined.

### Quantitative real-time PCR (qPCR)

Total RNA was prepared using TRIzon Reagent (CWBIO, China; Cat. CW0580S) according to the supplier's guidelines. Subsequently, one microgram of total RNA was reverse-transcribed to generate cDNA. The qPCR was performed with the 2×SYBR Green qPCR Mix Kit (Shandong Sparkjade Biotechnology Co., Ltd., Cat. AH0101) and gene-specific primers (sequences listed in Table S1). Relative mRNA abundance for each target was calculated and expressed relative to β-actin.

### Flow cytometry analysis of macrophage polarization markers

RAW 264.7 macrophages were collected and reconstituted in phosphate-buffered saline (PBS). Then, the Fc receptors were blocked by treating with CD16/32 antibody (Elabscience Biotechnology Co., Ltd., Cat. E-AB-F0997A) for 10 min. For polarization analysis, M1 macrophages were stained with fluorescein isothiocyanate (FITC)-conjugated CD80 antibody (Proteintech, Cat. FITC-65076), while M2 macrophages were labeled with FITC-conjugated CD206 antibody (Elabscience, Cat. E-AB-F1135C). Antibody labeling was conducted under light-protected conditions for 30 min. A BD FACSCelesta flow cytometer was employed for the analysis.

### Nitric oxide (NO) assay

Nitrite concentrations were analyzed using the Griess method, involving the combination of 50 μL of supernatant with equal volumes of Griess reagents I and II (Beyotime, Cat. S0021). The absorbance was recorded at 540 nm, and data were normalized to LPS group.

### Macrophage migration assay

RAW 264.7 macrophages were cultured until reaching 90-95% confluency. After a linear scratch was introduced into the cell monolayer, the cells were treated with CMA in 1% FBS-containing medium, followed by exposed to LPS (1 μg/mL). Scratch images were acquired after wounding (0 h) and again at 24 h under a microscope. Cell motility was quantified in ImageJ by calculating migration based on the remaining gap width (residual scratch width).

### Analysis of glucose consumption and lactate production

Supernatants were collected from M1-polarized macrophages treated with or without CMA after 24 h of culture. Glucose consumption was assayed with the Beyotime kit (Cat. S0201) following the recommended procedure. Briefly, 5 μL of supernatant was combined with 185 μL of reaction solution and subjected to 95°C heating, and subsequently measured at 630 nm. Glucose levels were calculated against a standard curve.

Lactate was determined by the CheKine™ Micro Lactate Assay Kit (Abbkine, Cat. KTB1100). Supernatant samples (50 μL) were added into 50 μL lactate detection working solution for 30 min, and subsequently measured at 450 nm. The calculation of lactate concentrations was performed by reference to a standard curve.

### Enzymatic activity assays of HK, PK, LDH and PDH

Hexokinase (HK) Activity Assay Kit (Cat. BC0740), Pyruvate Kinase (PK) Activity Assay Kit (Cat. BC0540), Lactate Dehydrogenase (LDH) Activity Assay Kit (Cat. BC0680) and Pyruvate Dehydrogenase (PDH) Activity Assay Kit (Cat. BC0380) were supplied by Beijing Solarbio Science & Technology Co., Ltd. (Beijing, China). Briefly, cells were lysed using extraction buffer. Cell lysates were then homogenized by sonication under the following conditions: 3-second pulses at 200 W with 10-second intervals, repeated for 30 cycles on ice. After centrifugation of the samples, the supernatants were collected and mixed with kit-specific reaction reagents and heated at 37°C for the manufacturer-specified durations. Optical density (OD) was monitored using a UV-Vis spectrophotometer (PerkinElmer, MA, USA) at 340 nm (HK and PK), 450 nm (LDH), and 605 nm (PDH). Lysate protein levels were determined with a BCA Protein Assay Kit (Beyotime, Cat. P0012), and enzyme activities were calculated on a per-protein basis and reported as fold differences compared with the control groups.

### Measurement of intracellular ATP levels

Cellular ATP concentrations were quantified by an ATP Detection Assay Kit (Beyotime, Cat. S0026). Briefly, each well received 100 μL of ATP assay working solution, followed by a 10-min dark incubation. Chemiluminescence was determined with a multimode microplate reader (Thermo Fisher Scientific). Luminescence values were expressed as relative ATP levels (%) compared to untreated controls.

### Seahorse analysis

RAW 264.7 macrophages were washed and incubated in Seahorse XF assay medium at 37°C in a non-CO₂ incubator for 1 h before measurements. For the mitochondrial stress test, assay medium was fortified with glucose (10 mM), L-glutamine (2 mM), and sodium pyruvate (1 mM). Oxygen consumption rate (OCR) measurements were obtained under basal conditions and following stepwise compound delivery: oligomycin (1 μM), FCCP (1.5 μM), and rotenone/antimycin A (1 μM). Seahorse Wave software was used to extract respiratory indices, including basal and maximal respiration, proton leak, and ATP-linked respiration. For the glycolysis stress assay, cells were incubated in assay medium fortified with L-glutamine (2 mM). Extracellular acidification rate (ECAR) was monitored before and after application of glucose (10 mM), oligomycin (1 μM), and 2-deoxyglucose (2-DG, 50 mM), from which glycolysis and glycolytic capacity were detected.

### siRNA transfection

BNIP3 was knocked down in RAW 264.7 macrophages using small interfering RNAs (siRNAs). Two independent siRNAs targeting mouse Bnip3 were used: siRNA1 (sense 5'-GCACAGCUACUCUCAGCAUTT-3') and siRNA2 (sense 5'-GGACGAAGUAGCUCCAAGATT-3'). Cells were transfected with siRNAs using Lipofectamine 3000 (Thermo Fisher Scientific) as recommended by the manufacturer. Validation of knockdown efficiency was carried out by immunoblotting. After transfection, cells were permitted to recover before CMA pretreatment (20 μM, 1 h) and subsequent LPS treatment (1 μg/mL, 24 h).

### Mitochondrial staining and determination of mitophagy flux

Mitochondrial staining was performed using Mito-Tracker Green (MTG; Beyotime, Cat. C1996). Cells were seeded in confocal dish (NEST Biotechnology, Jiangsu, China) and incubated with the dye (200 nM) for 30 min. Fluorescence imaging was conducted using LSM800 microscope (Carl Zeiss, BW, Germany) with excitation/emission wavelengths set to 488/530 nm. Mitochondrial fluorescence intensity corresponding to individual cells was quantified using ImageJ.

Mitophagic flux was assessed using a previously described method 28. Briefly, cells were treated with or without the bafilomycin A1 (Baf A1, 200 nM) prior to mitochondrial staining. Mitophagic flux was calculated by dividing the Mito-Tracker Green fluorescence intensity in Baf A1-treated cells by that in untreated cells, measured *via* flow cytometry.

### Mitochondrial membrane potential (MMP) assays

The JC-1 probe (Beyotime, Cat. C2006) was used to determine MMP. Briefly, a 20-min incubation of cells with freshly prepared JC-1 working solution was performed. Fluorescence signals were detected using an EVOS FL automated imaging system and a multimode microplate reader (Thermo Fisher Scientific). JC-1 monomers were detected at excitation/emission wavelengths of 490/530 nm, while aggregates were detected at 525/590 nm. Fluorescence ratios (aggregate/monomer) were calculated to quantify relative MMP.

### Western blot

Total proteins were harvested by RIPA lysis buffer fortified with protease inhibitor cocktail (AbMole, USA; Cat. M5293) on ice for 30 min. Protein samples (equal amounts) were mixed with 5× loading buffer, subjected to 99°C for 10 min for denaturation, and resolved by SDS-PAGE. Following electrophoresis, proteins were wet-transferred onto PVDF membranes (Millipore, MA, USA) using a Bio-Rad transfer apparatus (CA, USA). Membranes were subjected to blocking in 5% nonfat milk for 2 h and subsequently cultured with the indicated primary antibodies and HRP-labeled secondary antibodies. Immunoreactive bands were visualized using ECL substrate (Millipore, Cat. WBKLS0500). The following primary antibodies were used: LAMP1(Servicebio, Cat. GB112949), Parkin (Servicebio, Cat. GB113802), LC3B (Proteintech, Cat. 14600-1-AP), BNIP3 (Proteintech, Cat. 68091-1-lg), PINK1 (BIOSS, Cat. bsm-51265m), TOMM20 (CUSABIO, Cat. CSB-PA618983ESR2HU, https://www.cusabio.com/), β-actin (Servicebio, Cat. GB11001), GAPDH (Servicebio, Cat. GB12002).

### Co-localization analysis of mitochondria and lysosomes

Mitochondria and lysosomes were labeled using MTG and LysoTracker Red (LTR; Beyotime, Cat. C1046), respectively. Cells were incubated with MTG (200 nM) for 30 min, followed by LTR (50 nM) for 20 min. Using excitation/emission wavelengths of 577/590 nm, LTR fluorescence was visualized with an LSM800 microscope (Carl Zeiss). Pearson's correlation and overlap coefficient were calculated to quantify the spatial overlap between mitochondria and lysosomes using ImageJ, reflecting the extent of mitophagy.

### Transmission electron microscopy (TEM) assay

Following fixation with 1% osmium tetroxide for 1.5 h, the cells were dehydrated by sequential immersion in graded ethanol (30%, 50%, 70%, 80%, and 95%). For contrast enhancement, ultrathin sections were stained with uranyl acetate for 10 min and subsequently with lead citrate for 10 min. Imaging was performed using a TEM (JEOL JEM-1400Plus, JEOL, Japan) operated at 80 kV.

### Preparation of macrophage membrane-biomimetic nanoparticles

Macrophage membranes were isolated from RAW 264.7 cells using a differential centrifugation protocol 29. Briefly, cells were transferred into TM buffer (10 mM Tris-HCl, 1 mM MgCl_2_, pH 7.4) and maintained on ice for 20 min. Cell lysis was achieved by homogenizing the suspension with a glass Dounce homogenizer on ice. Nuclei and unbroken cells were pelleted by centrifuging the lysate at 2500 rpm (4°C, 10 min) after adjusting its sucrose concentration to 0.25 M with 1 M sucrose-TM buffer. The supernatant was collected, homogenized again, and centrifuged under the same conditions. The resulting supernatant was further centrifuged at 12000 rpm (4°C, 30 min) to pellet membrane fractions. The membrane pellet was washed with 0.25 M sucrose-TM buffer and centrifuged at 12000 rpm (4°C, 20 min). Extrusion of purified membranes was performed 10 times through a polycarbonate membrane (400 nm pore size; Whatman, MA, USA) using a mini-extruder (Avanti Polar Lipids, USA) to ensure uniformity. Total membrane yield was estimated based on protein content.

MM@CMANP or MM-biomimetic rhodamine B-loaded NP (MM@RBNP) were prepared using a liposome extruder. Briefly, Macrophage membranes and CMANP were combined in equal masses. The mixture was processed with a probe sonicator at 120 W for 3 min, followed by sequential extrusion through a polycarbonate membrane (200 nm pore size) using a mini-extruder for 10 passes. Filtration of the MM@CMANP suspension through a 0.22 μm sterile syringe filter was performed, and the product was refrigerated at 4°C until needed. Protein profiles of macrophage membranes were verified *via* SDS-PAGE and coomassie brilliant blue staining. Markers of macrophage membrane including CD11b (zenbio, R380675), TNFR2 (zenbio, R300343), and CCR2 (zenbio, R380703), were detected by western blot.

### *In vitro* release assay of drugs

A dialysis setup was employed to determine the *in vitro* release behavior of the nanomedicine formulations. Briefly, the nanomedicines solution was loaded into a dialysis membrane (MWCO: 3500 Da) and submerged in 50 mL of PBS (pH 7.4). Sampling was performed at predetermined time points (0, 1, 2, 4, 8, 16, 24, 48, and 72 h) by removing 5 mL of supernatant and replacing it with fresh PBS. Throughout the experiment, the system was maintained at 37°C with continuous orbital agitation at 50 rpm. Drug concentration in collected samples was measured by UV-Vis spectrophotometry (PerkinElmer) at 289 nm.

### Animal and treatment

Male C57BL/6 mice (8 weeks old) were obtained from the Guangdong Medical Laboratory Animal Center and acclimatized for 1 week prior to experiments. All *in vivo* protocols were reviewed and authorized by the Animal Ethics Committee of Guangdong University of Technology (GDUTX-S2023025). Administration of 3% dextran sulfate sodium (DSS; Yeasen, Shanghai, China; Cat. 60316ES60) in drinking water was used to induce the IBD model. As illustrated in Figure 7A, during DSS induction, treatment groups received 5-aminosalicylic acid (5-ASA; 100 mg/kg, oral gavage), CMANP or MM@CMANP (4 mg/kg, intravenous injection *via* the tail vein). Body weight, fecal consistency, and occult blood (Solarbio; Cat. BC8271) were monitored daily. At the experimental endpoint, mice were euthanized. Serum was separated from collected blood by centrifuging at 3,000 × g for 10 min. Serum levels of TNF-α (Neobioscience Technology Co, Ltd., Shenzhen, China; Cat. EMC102a.96), IL-6 (Jonlnbio, Shanghai, China; Cat. JL20268), IL-1β (Jonlnbio; Cat. JL18442), and IL-10 (Neobioscience Technology Co, Ltd.; Cat. EMC005.96) were detected using ELISA kits following the supplier's protocol. Colon tissues were excised and photographed. Subsequently, hematoxylin and eosin (HE) staining and Alcian blue-periodic acid Schiff's (AB-PAS) staining were performed for histopathological analysis.

### *In vivo* biodistribution of nanomedicines

The biodistribution of RBNP or MM@RBNP was investigated in an IBD mouse model. On day 8, RBNPs or MM@RBNPs were administered *via* tail vein injection. Following euthanasia at 2 h after injection, the heart, lungs, kidneys, spleen, liver, and colon were collected from the mice. *Ex vivo* fluorescence images were captured with an animal *in vivo* imaging system (PerkinElmer IVIS Lumina Series III) at 546 nm excitation.

### Immunohistochemistry (IHC) and immunohistofluorescence (IHF) assay

IHC and IHF assay were performed as previously described 30. Briefly, tissue sections from the colon were exposed to 3% hydrogen peroxide for 25 min. Tissue sections were first permeabilized using 0.2% Triton X-100, followed by blocking with 3% bovine serum albumin (BSA). The sections were exposed to primary antibodies and maintained at 4°C overnight. After washing, sections were exposed to secondary antibodies for 50 min at ambient temperature. For IHC, 3,3′-diaminobenzidine (DAB) served as the chromogen, with hematoxylin providing nuclear counterstaining. For IHF, sections were counterstained with 4',6-diamidino-2-phenylindole (DAPI) to reveal nuclei. Imaging of sections was performed on a ZEISS Axio Scan.Z1 automated slide scanner with Zen Blue software. The following primary antibodies were used: Occludin (Servicebio; Cat. GB111401), CD80 (Proteintech; Cat. 14292-1-AP), CD206 (Servicebio; Cat. GB113497).

### Colonic lamina propria macrophage isolation and flow cytometry

Colonic lamina propria cells were isolated from mouse colons by sequential EDTA/DTT stripping and enzymatic digestion. Briefly, colons were excised and then cut open along the longitudinal axis. After rinsing with ice-cold PBS, the tissues were minced. Tissues were incubated in HBSS containing 5 mM EDTA, 2 mM DTT, 10 mM HEPES, 1% penicillin-streptomycin, and 5% FBS with shaking (200 rpm) for 20 min. After washing, the remaining tissue was digested in RPMI-1640 containing collagenase IV (1.5 mg/mL) and DNase I (20 U/mL) (plus 1% penicillin-streptomycin and 5% FBS) at 200 rpm for 30 min. Cell suspensions were filtered through a 70-μm strainer and enriched by 40%/80% Percoll gradient centrifugation (600×g, 20 min). Collection, washing, and resuspension of cells from the interface were performed for subsequent staining.

For flow cytometry, cells were stained with APC-anti-F4/80 and PE-anti-CD11b to gate lamina propria macrophages (F4/80⁺CD11b⁺). Macrophage polarization was then assessed by staining with FITC-anti-CD80 (M1-like) and PE/Cy7-anti-CD206 (M2-like).

### Statistical analysis

Statistical analyses were performed using GraphPad Prism 9.5 software (San Diego, CA, USA). All data were derived from at least three independent experimental replicates or three independent biological samples. Group differences were analyzed using Student's t-test, one-way analysis of variance (ANOVA), or two-way ANOVA, as appropriate.

## Results

### CMA suppresses M1 macrophages polarization and promotes macrophages M2 polarization

**Figure 1:**
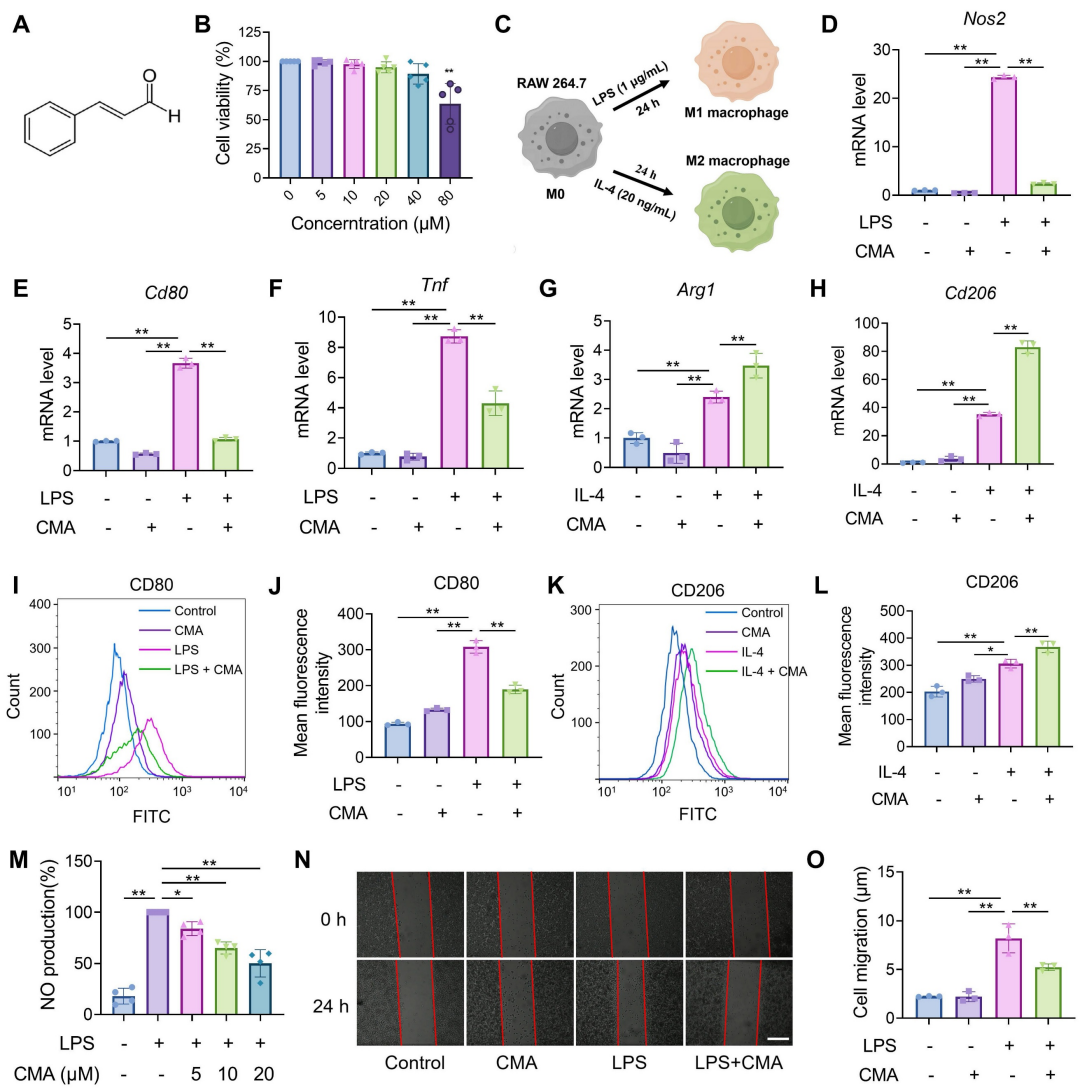
** CMA skews macrophage polarization toward M2 while attenuating M1 differentiation. (A)** Chemical structural formula of CMA. **(B)** RAW 264.7 cells were treated with CMA (0-80 μM) for 24 h, followed by incubated with MTT (0.5 mg/mL) for 4 h (n = 5). **(C)** Schematic illustration of macrophage polarization mediated by different inducers. **(D-F)** The mRNA levels of M1 markers (*Nos2, Cd80,* and* Tnf*) in RAW 264.7 macrophages pretreated with CMA (20 μM) for 1 h, followed by co-treatment with LPS (1 µg/mL) for 24 h (n = 3). **(G, H)** The mRNA levels of M2 markers (*Arg1* and* Cd206*) in RAW 264.7 macrophages pretreated CMA (20 μM) for 1 h, followed by co-treatment with IL-4 (20 ng/mL) for 24 h (n = 3). **(I, J)** Flow cytometry analysis and quantification of CD80 protein levels in M1 macrophages treated with or without CMA (20 μM) for 24 h (n = 3). **(K, L)** Flow cytometry analysis and quantification of CD206 in M2 macrophages treated with or without CMA (20 μM) for 24 h (n = 3). **(M-O)** Effects of CMA treatment on NO levels (n = 4) and cell migration (n = 3) in RAW 264.7 macrophages. Scale bar: 100 μm. Data presented as mean ± SD, *P < 0.05, **P < 0.01.

The progression of IBD is accompanied by abnormal macrophage polarization, and modulating macrophage polarization represents a viable approach for treating the disease 6. Therefore, the effect of CMA on regulating the polarization of macrophages toward M1/M2 phenotypes was investigated *in vitro*. First, it was confirmed that the safe dose of CMA (Figure 1A) for RAW 264.7 macrophages was below 40 μM (Figure 1B), and a dose of 20 μM was selected for further investigation. Polarization of RAW 264.7 macrophages into M1 or M2 phenotypes was achieved by stimulation with LPS or IL-4, respectively (Figure 1C). CMA treatment significantly downregulated the mRNA levels of M1 macrophage markers, including *Nos2*, *Cd80*, and *Tnf* (Figure 1D-F), and inhibited the protein expression of CD80 (Figure 1I, J). In contrast, CMA significantly upregulated the mRNA levels of M2 macrophage markers, including *Arg1* and *Cd206* (Figure 1G, H), and increased the protein expression of CD206 (Figure 1K, L). To further investigated the inhibitory effect of CMA on M1 macrophages, NO production and cell migration in LPS-induced RAW 264.7 macrophages were assessed. Compared to the LPS-induced M1 macrophages, CMA treatment significantly decreased NO production (Figure 1M) and inhibited M1 macrophage migration (Figure 1N, O).

### CMA reprograms the metabolism profiles of M1 macrophages

**Figure 2:**
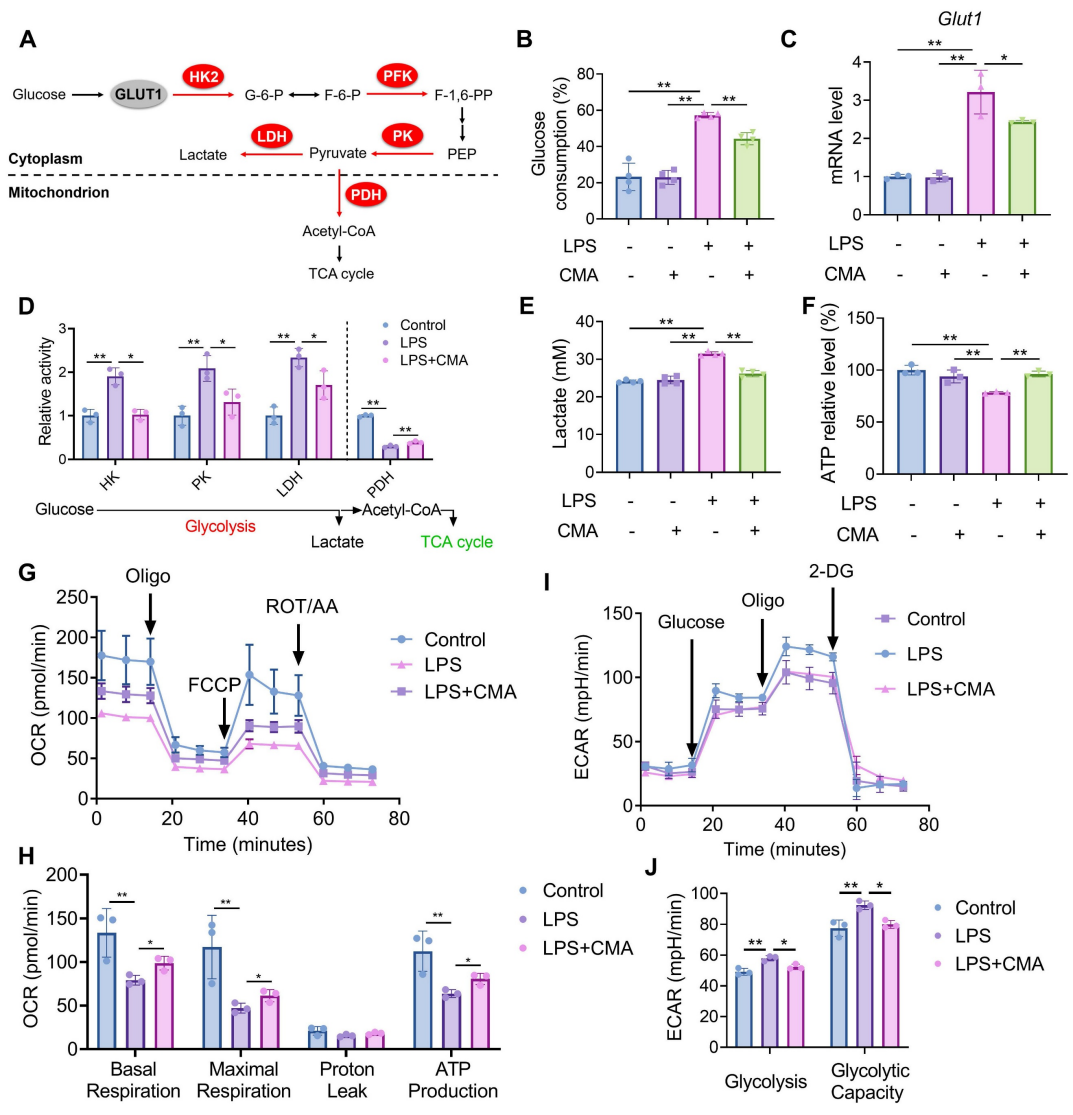
** CMA reprograms the metabolic profiles of M1 macrophages.** RAW 264.7 macrophages were pretreated with CMA (20 μM) for 1 h, followed by co-treatment with LPS (1 µg/mL) for 24 h. **(A)** Schematic representation of the glucose metabolism pathway. **(B)** Glucose consumption was measured by analyzing glucose levels in the supernatant of RAW 264.7 macrophages (n = 4). **(C)** The mRNA level of *Glut1* in RAW 264.7 macrophages (n = 3). **(D)** Enzymatic activities of HK, PK, LDH, and PDH in RAW 264.7 macrophages (n = 3). **(E, F)** Lactate production in the supernatants (n = 4) and intracellular ATP levels (n = 3) of RAW 264.7 macrophages. **(G, H)** OCR traces and quantification of mitochondrial respiration parameters in the Seahorse mitochondrial stress test (n = 3). **(I, J)** ECAR traces and quantification of glycolysis and glycolytic capacity in the Seahorse glycolysis stress test (n = 3). Data presented as mean ± SD, *P < 0.05, **P < 0.01.

M1 macrophages exhibit a metabolic phenotype characterized by enhanced aerobic glycolysis and reduced oxidative phosphorylation 31. To evaluate whether CMA modulates the metabolic profiles of M1 macrophages, changes in glucose consumption was assessed first. Compared to M0 macrophages, M1 macrophages demonstrated increased glucose demand, which was significantly reduced upon CMA treatment (Figure 2B). Given the crucial role of the glucose transporter GLUT1 in glucose uptake by M1 macrophages 32, we investigated whether CMA reduces glucose uptake by inhibiting GLUT1. As shown in Figure 2C, CMA treatment decreased GLUT1 mRNA expression in M1 macrophages. The activity of key enzymes mediating glycolysis and mitochondrial oxidative phosphorylation (Figure 2A) was further evaluated. It was observed that Upon LPS challenge, macrophages exhibited upregulated glycolysis, as evidenced by increased HK, PK, and LDH activities. Conversely, the activity of PDH, a key regulator of the TCA cycle, was diminished. CMA treatment significantly revised the activity of these enzymes in M1 macrophages (Figure 2D). CMA treatment also improved the increased lactate production and decreased ATP levels in M1 macrophages (Figure 2E, F). We further performed Seahorse extracellular flux analysis to visualize metabolic reprogramming in real time. In the mitochondrial stress test, LPS markedly reduced OCR throughout the assay, accompanied by significant decreases in basal respiration, maximal respiration, and ATP-linked respiration compared with the control group, whereas CMA treatment partially restored these mitochondrial respiratory parameters (Figure 2G, H). In parallel, glycolysis stress testing showed that LPS elevated ECAR after glucose/oligomycin injection (increased glycolysis and glycolytic capacity), and these glycolytic responses were significantly attenuated by CMA (Figure 2I, J). Collectively, these findings suggest that CMA reverses the metabolic reprogramming of M1 macrophages by inhibiting their metabolic profiles.

### CMA improves mitochondrial homeostasis in M1 macrophages

**Figure 3:**
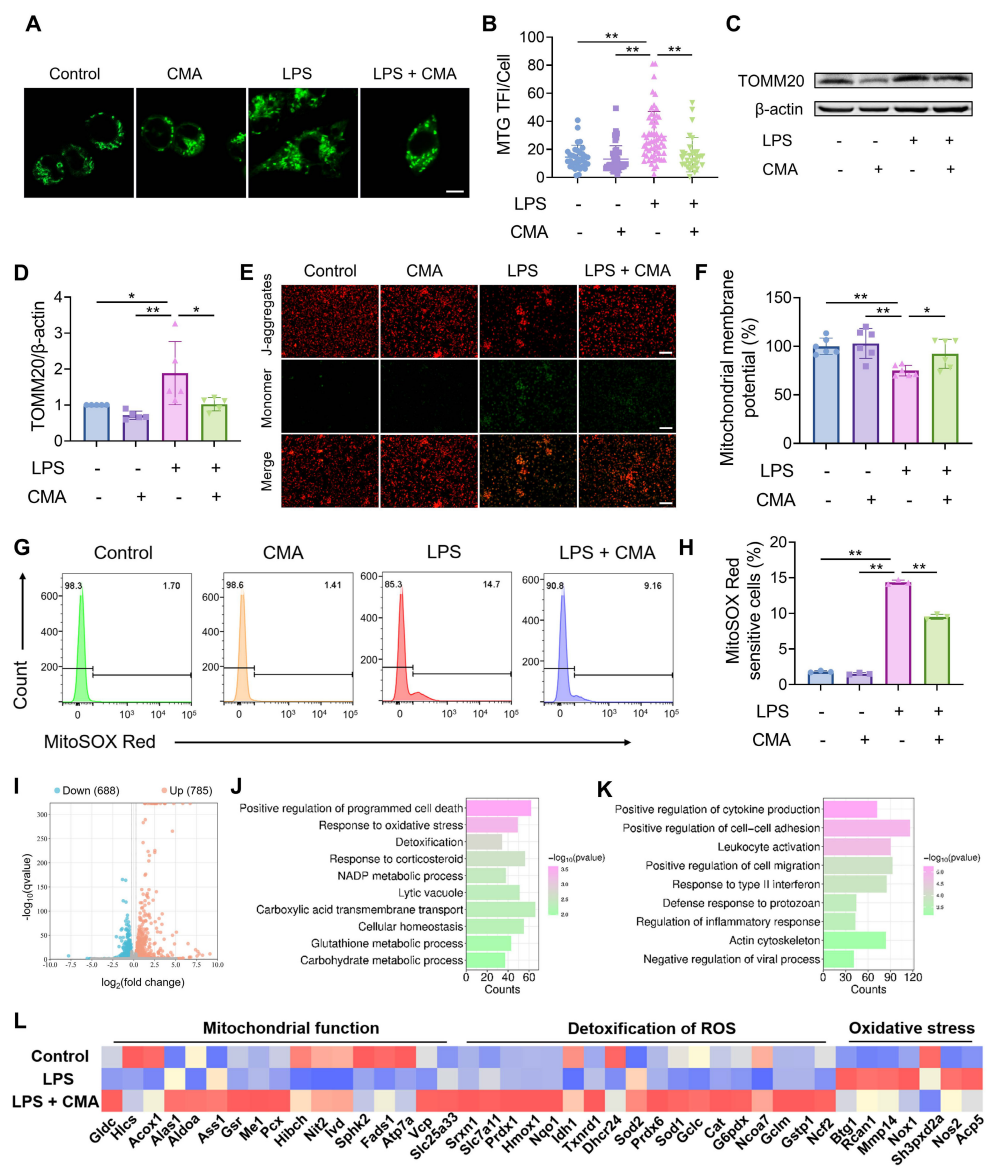
** CMA improves mitochondrial homeostasis in M1 macrophages.** Following a 1-h pretreatment with CMA (20 μM), RAW 264.7 macrophages were stimulated with LPS (1 µg/mL) for 24 h. **(A, B)** Representative images and relative quantification of mitochondria from MTG-labeled RAW 264.7 macrophages. Quantification was performed by counting mitochondrial fluorescence in all cells in four to eight random fields, respectively. Scale bar: 5 μm. **(C, D)** Representative immunoblot bands and quantification of the mitochondrial marker TOMM20 in RAW 264.7 macrophages (n = 5). **(E, F)** Fluorescence microscopy and fluorescence microplate reader analysis of JC-1-labeled MMP in RAW 264.7 macrophages. Scale bar: 100 μm. **(G, H)** Flow cytometry and quantification of MitoSOX Red-labeled mtROS in RAW 264.7 macrophages (n = 3). **(I)** Volcano plot shows DEGs in the LPS group vs LPS + CMA group. **(J)** GO enrichment analysis of upregulated DEGs in the LPS group vs LPS + CMA group. **(K)** GO enrichment analysis of downregulated DEGs in the LPS group vs LPS + CMA group. **(L)** Heat map of DEGs related to mitochondrial function, ROS detoxification, and oxidative stress pathways in control, LPS and LPS + CMA groups (n = 3). Data presented as mean ± SD, *P < 0.05, **P < 0.01.

To assess whether the altered metabolic profiles of mitochondria in M1 macrophages is associated with mitochondrial dysfunction, Mito-Tracker Green (MTG), a dye that does not depend on MMP, was used to stain with macrophages. Significant increase in mitochondrial content was found in M1 macrophages, which was reduced by CMA treatment (Figure 3A, B). A similar pattern was observed by detecting the mitochondrial marker TOMM20 (Figure 3C, D). We hypothesized that the increased mitochondrial content in M1 macrophages is due to the accumulation of dysfunctional mitochondria, accompanied by a loss of MMP. Figure 3E, F showed a significant decrease in the aggregates/monomer ratio in M1 macrophages compared to control cells, indicating loss of MMP. Notably, this depolarization was substantially attenuated by CMA administration. Excessive production of reactive oxygen species (ROS) has been shown to cause loss of MMP and mitochondrial dysfunction, which is essential for maintaining the pro-inflammatory state of M1 macrophages 33. In M1 macrophages, both ROS (Figure S1A, C) and mitochondrial ROS (mtROS) (Figure 3G, H and Figure S1B, D) levels were significantly elevated, and these abnormalities improved after treatment CMA.

To further investigate the genes regulated by CMA in M1 macrophages, RNA sequencing analysis was performed. After CMA treatment, 785 genes were upregulated, and 688 genes were downregulated (Figure 3I). GO enrichment analysis was performed on these DEGs. The results showed that the upregulated DEGs were mainly involved in biological processes such as response to oxidative stress, detoxification, and NADP metabolic process (Figure 3J). The downregulated DEGs were mainly concentrated in pathways such as positive regulation of cytokine production, leukocyte activation, and regulation of inflammatory response (Figure 3K). Notably, genes related to mitochondrial function, such as those involved in oxidative phosphorylation (*Atp7a*, *Vcp*, *Slc25a33*, etc) and ROS detoxification (*Prdx1*, *Txnrd1*, *Sod1*, etc), were downregulated in M1 macrophages compared to M0 macrophages. In contrast, genes related to oxidative stress (*Mmp14*, *Nox1*, *Nos2*, etc) were upregulated. This dysregulation was ameliorated by CMA treatment (Figure 3L). Overall, these observations imply that CMA intervenes in mitochondrial metabolism by maintaining mitochondrial homeostasis, thereby suppressing M1 macrophage polarization.

### CMA improves mitochondrial homeostasis of M1 macrophages *via* promoting BNIP3-mediated mitophagic flux

**Figure 4:**
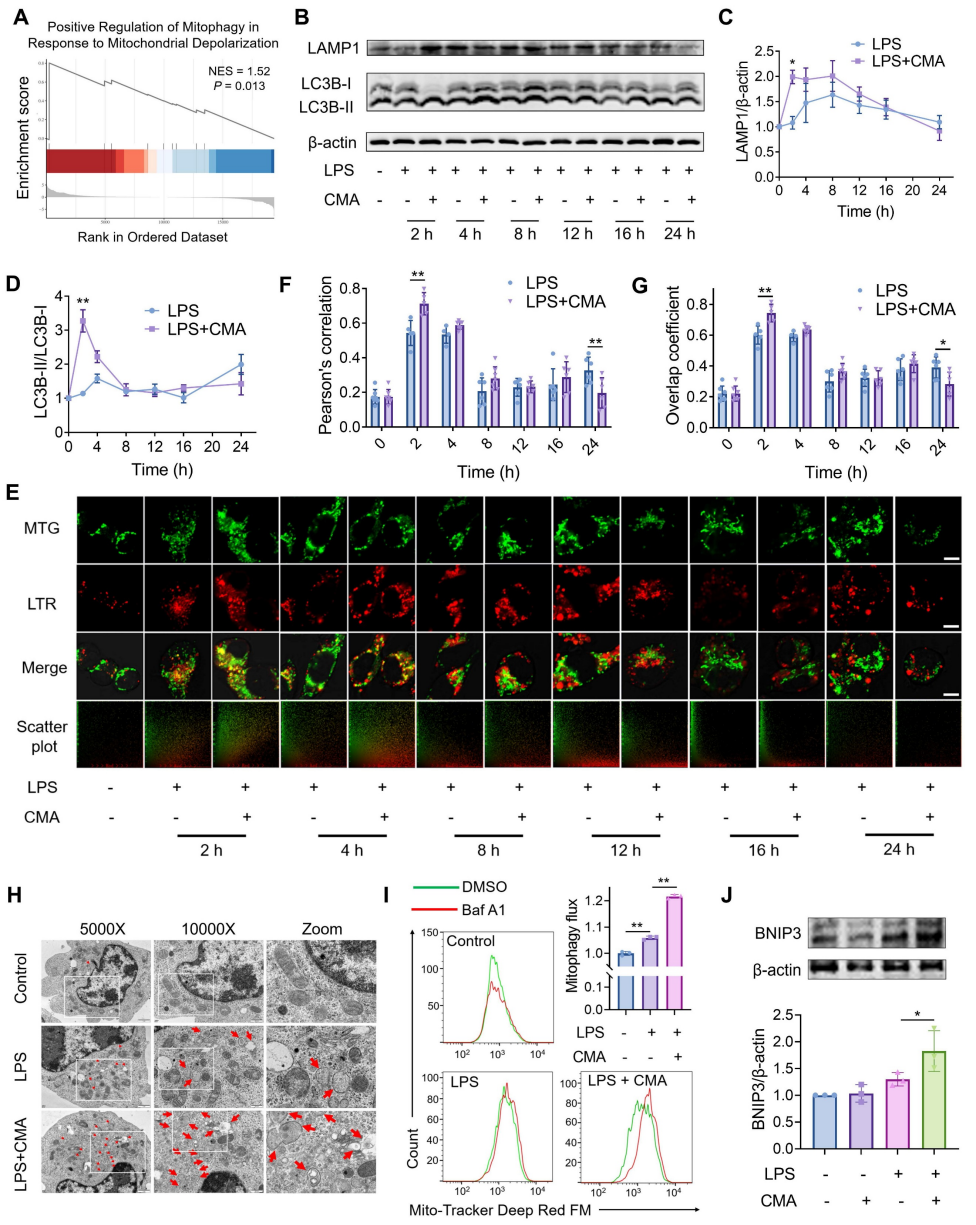
** CMA improves mitochondrial homeostasis of M1 macrophages *via* promoting BNIP3-mediated mitophagic flux. (A)** GSEA revealed significant enrichment of the pathway for positive regulation of mitophagy in response to mitochondrial depolarization in CMA-treated M1 macrophages. **(B-D)** Representative immunoblot bands and quantification of the lysosomal marker LAMP1 and the autophagosome marker LC3B in M1 macrophages treated with or without CMA for different time gradients (n = 3). **(E-G)** Representative images of MTG-labeled mitochondria and LTR-labeled lysosomes, and analysis of the degree of co-localization of these two organelles. The spatial association between mitochondria and lysosomes was assessed by quantifying four to seven randomly fields. Scale bar: 5 μm. **(H)** Representative TEM analysis in M1 macrophages treated with or without CMA (20 μM) for 2 h. **(I)** Flow cytometry and quantification of the mitophagy flux in M1 macrophages treated with or without CMA (20 μM) for 2 h. Mitophagy flux was defined as the inhibitory portion of Baf A1 by staining mitochondria using Mito-Tracker Deep Red FM (n = 3). **(J)** Representative immunoblot bands and quantification of BNIP3 in M1 macrophages treated with or without CMA for 2 h (n = 3). Data presented as mean ± SD, *P < 0.05, **P < 0.01.

Mitophagy is a crucial process for cells to maintain mitochondrial homeostasis 34. GSEA (Figure 4A) and KEGG pathway (Figure S2A) enrichment indicated that CMA modulates mitophagy in M1 macrophages. Therefore, we first measured the number of autophagosomes in M1 macrophages treated with CMA for different time points using MDC staining. After 2 hours of CMA treatment, the number of autophagosomes in M1 macrophages significantly increased, then gradually decreased (Figure S3A, B). During the formation of autophagosomes, the LC3-I protein undergoes ubiquitin-like modification and is converted to its autophagosomal membrane-bound form LC3-II. LAMP1, a key glycosylated protein involved in lysosome biogenesis, serves as an important marker of lysosomes. Time-dependent CMA treatment of M1 macrophages showed a significant increase in both autophagosome formation and lysosome biogenesis after 2 hours (Figure 4B-D). We further used MTG and LTR to label mitochondria and lysosomes, respectively, and assessed the extent of mitophagy by examining the co-localization between the two. Consistent with the previous results, at 2 hours post CMA treatment, the Pearson correlation and overlap coefficients between mitochondria and lysosomes were significantly higher in the CMA-treated group than in the M1 macrophages group, indicating that CMA enhanced mitophagy in M1 macrophages in a short time frame (Figure 4E-G). TEM was performed to directly observe changes in autophagosomes or autolysosomes. Compared to M0 macrophages, M1 macrophages exhibited the formation of vesicles with single or multi-layered membranes, some of which contained mitochondria, displaying typical features of mitophagy. In contrast, CMA-treated M1 macrophages produced more vesicles containing mitochondria (red arrows), indicating that CMA promotes mitophagy in M1 macrophages (Figure 4H). Furthermore, we confirmed that CMA increased mitophagic flux in M1 macrophages by using the lysosomal inhibitor Baf A1 (Figure 4I).

The regulation of mitophagy involves both ubiquitin-dependent and non-ubiquitin-dependent pathways, and the ubiquitin-dependent PTEN-induced putative kinase 1 (PINK1)/Parkin pathway is typically activated in response to mitochondrial damage 35. However, CMA treatment did not affect the PINK1/Parkin pathway in M1 macrophages (Figure S3C-E). RNA sequencing revealed that CMA treatment upregulated *Bnip3* gene expression in M1 macrophages (Figure S2B), a key autophagic receptor in the non-ubiquitin-dependent pathway. Additionally, CMA treatment increased the protein expression of BNIP3 in M1 macrophages (Figure 4J). All these results indicate that CMA improves mitochondrial homeostasis in M1 macrophages by promoting BNIP3-mediated mitophagy.

### Genetic and pharmacological blockade of mitophagy reverses the inhibitory effect of CMA on M1 macrophage phenotype

**Figure 5:**
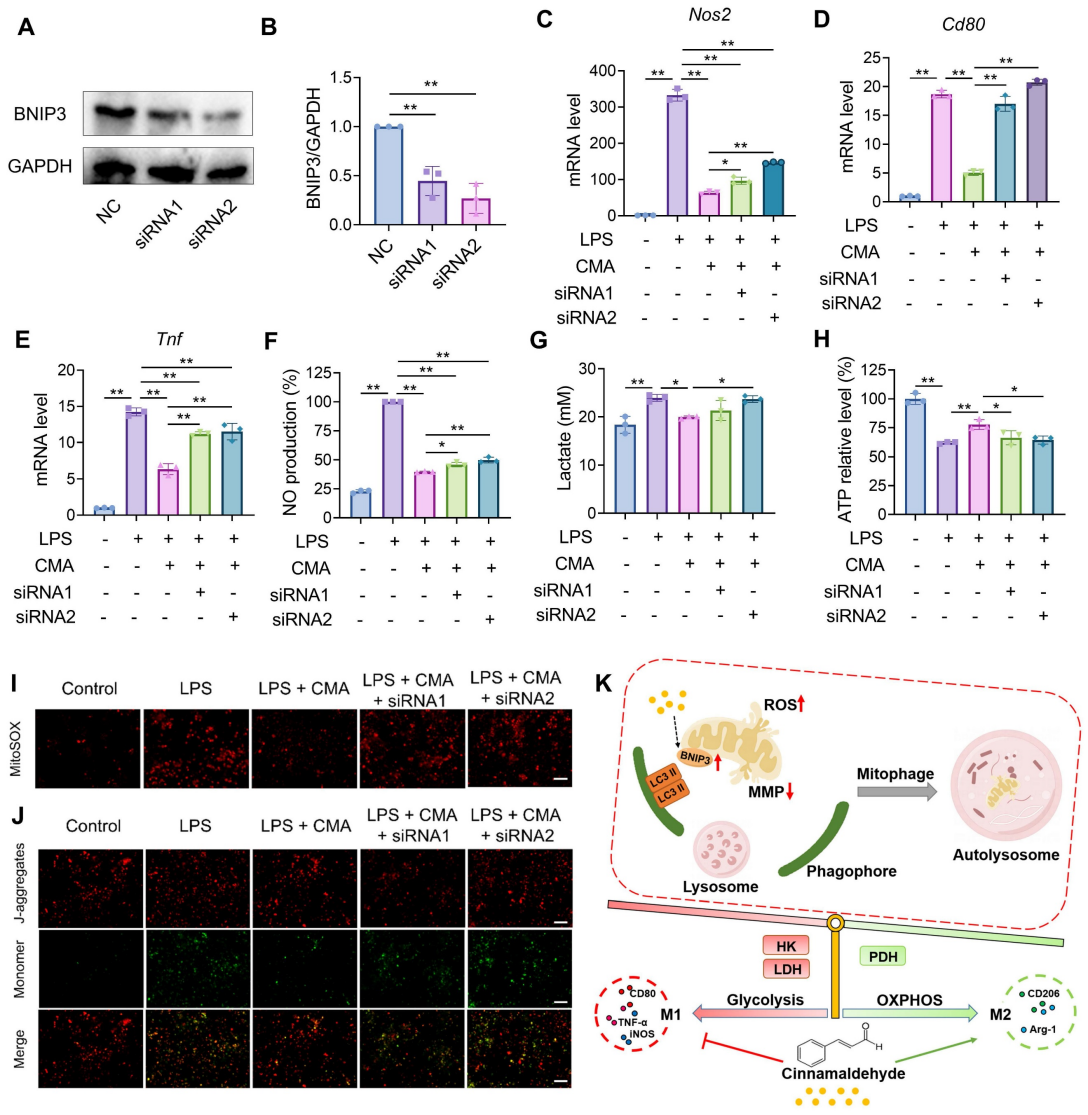
** Knockdown of BNIP3 reverses the suppressive effect of CMA on M1 markers and mitochondrial dysfunction.** A 1-h pretreatment with CMA (20 μM) was performed on RAW 264.7 macrophages, followed by 24-h exposure to LPS (1 µg/mL). **(A, B)** The efficiency of BNIP3 knockdown in RAW 264.7 cells (n = 3). **(C-E)** The mRNA level of *Nos2*, *Cd80*, and *Tnf* in RAW 264.7 macrophages (n = 3). **(F, G)** NO levels and lactate production in the supernatants of RAW 264.7 macrophages (n = 3). **(H)** Intracellular ATP levels of RAW 264.7 macrophages (n = 3). **(I, J)** Representative fluorescence images and quantitative analysis of mtROS detected by MitoSOX in RAW 264.7 macrophages. Scale bar: 100 μm. **(K)** Mechanism of action of CMA. CMA promotes BNIP3-mediated mitophagy to eliminate damaged mitochondria, thereby shifting macrophage energy metabolism toward oxidative phosphorylation and inhibiting macrophage polarization to the M1 phenotype. Data presented as mean ± SD, *P < 0.05, **P < 0.01.

To validate the requirement of mitophagy in CMA-mediated suppression of M1 polarization, we blocked lysosome-dependent autophagy flux using chloroquine (CQ; TargetMol, USA) or Baf A1 (MeilunBio, China), and genetically depleted the mitophagy receptor BNIP3 using two independent siRNAs (siRNA1 and siRNA 2). BNIP3 knockdown efficiency was confirmed by immunoblotting (Figure 5A, B). In LPS-stimulated RAW 264.7 macrophages, CMA markedly reduced *Nos2*, *Cd80* and *Tnf* expression as well as NO production. However, CQ or Baf A1 treatment and BNIP3 knockdown significantly attenuated these inhibitory effects (Figure 5C-F; Figure S4A-D). Consistently, CMA decreased lactate accumulation and restored intracellular ATP, but these metabolic benefits were blunted by CQ/Baf A1 and by BNIP3 knockdown (Figure 5G, H; Figure S4 E, F). At the mitochondrial level, CMA alleviated LPS-induced mitochondrial stress by improving MMP and reducing mtROS. Both pharmacological inhibition of autophagy flux and BNIP3 depletion partially abolished these improvements (Figure 5I, J; Figure S4G-J). The concordant pharmacological and genetic evidence demonstrates that CMA restores mitochondrial homeostasis and metabolic profiles in macrophages by promoting BNIP3-mediated mitophagy, thereby inhibiting macrophages from tilting to M1 phenotype (Figure 5K).

### Design of macrophage membrane (MM) biomimetic nano-delivery system for CMA

Although CMA has great potential in regulating macrophage polarization, its application *in vivo* is challenged by poor water solubility, low stability, and low oral bioavailability. Given the homing effect of macrophages to sites of inflammation, macrophage membrane-coated nanoparticles may target and accumulate at these sites. Specifically, chemokine receptors located on the macrophage membrane facilitate the recruitment of macrophage membrane-biomimetic nanoparticles to inflammatory lesions. Therefore, macrophage membrane-coated CMA-loaded nanoparticles (MM@CMANP) were designed. First, nanoprecipitation was employed to obtain CMA-loaded poly(lactic-co-glycolic acid) (PLGA) nanoparticles (CMANP). Dynamic light scattering (DLS) measurements showed that CMANP had an effective particle size of 129.7 nm, a polydispersity index (PDI) of 0.078 (Figure S5A), and a zeta potential of -14.68 mV (Figure S5B). It is generally accepted that a PDI of <0.3 indicates a relatively uniform particle size distribution 36. Additionally, the encapsulation and drug loading efficiencies of CMANP were 61.46% and 8.91%, respectively (Figure S5C). Stability testing showed that CMANP maintained its effective particle size and zeta potential over 28 days (Figure S5D, E), confirming the successful preparation of CMANP. MM@CMANP showed no appreciable change in hydrodynamic size over 3 days of incubation in mouse serum, indicating good serum stability under physiologically relevant conditions (Figure S5H).

**Figure 6:**
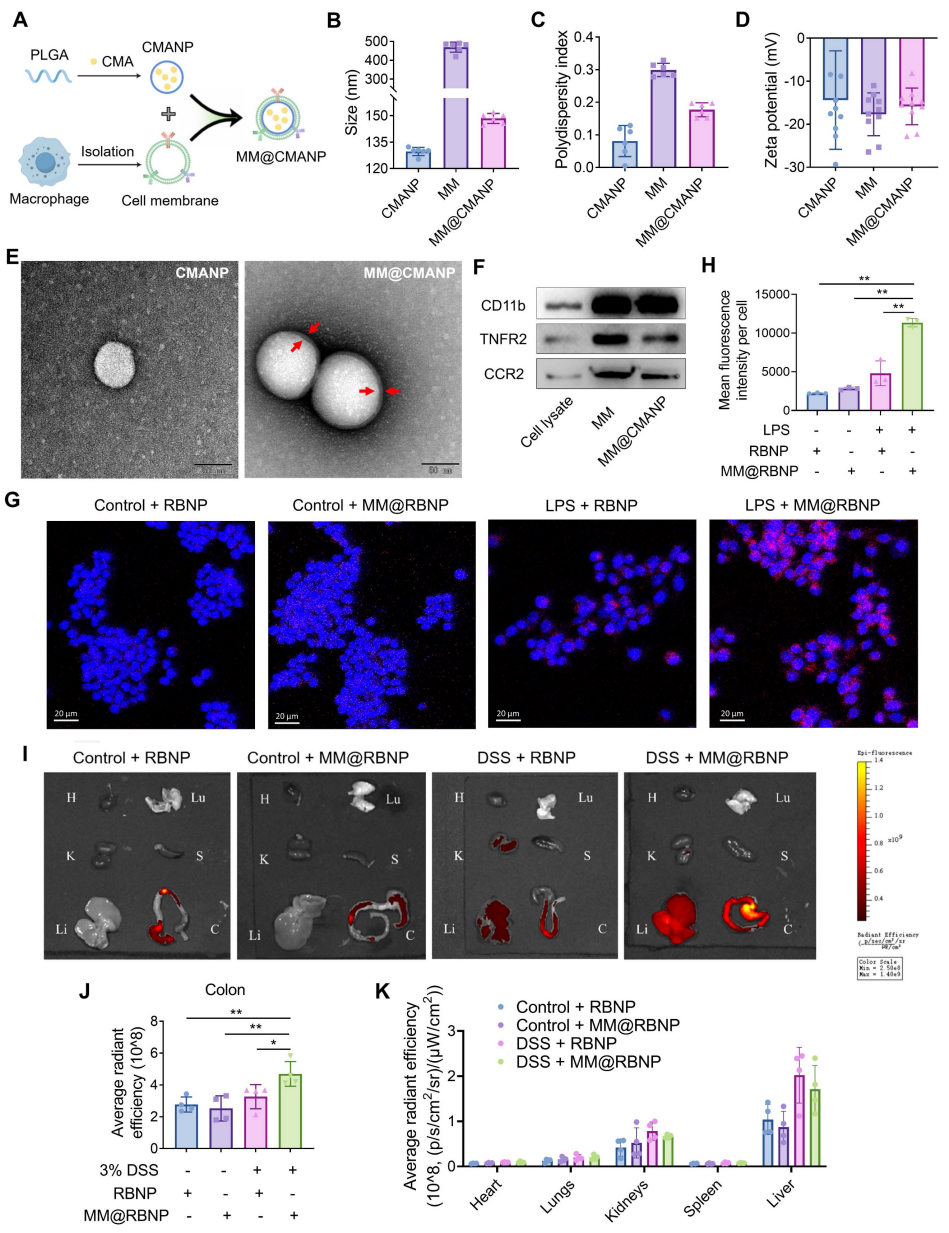
** Preparation, characterization and biodistribution of macrophage membrane biomimetic nanoparticles. (A)** Schematic illustration of the preparation of MM@CMANP. **(B-D)** Size, PDI and zeta potential of CMANP, MM and MM@CMANP analyzed by DLS, respectively. **(E)** TEM analysis of CMANP and MM@CMANP. Scale bar: 50 nm. **(F)** Western blot analysis of macrophage membrane proteins (CD11b, TNFR and CCR2) in RAW 264.7 cell lysate, MM, and MM@CMANP. **(G, H)** *In vitro* targeting was assessed by examining the internalization of MM@CMANP by RAW 264.7 cells. **(I-K)** *In vivo* targeting, biodistribution and quantification of RBNP and MM@RBNP in the colon and major organs of normal and IBD mice at 2 h post-intravenous administration. H, Lu, K, S, Li, C represent the heart, lung, kidney, spleen, liver and colon, respectively (n = 4). Data presented as mean ± SD, *P < 0.05, **P < 0.01.

To synthesize MM@CMANP, macrophage membranes were coated onto CMANP by an extrusion method (Figure 6A). The effective particle size of MM@CMANP was 18 nm larger than that of CMANP (Figure 6B), and the PDI and zeta potential were intermediate between CMANP and the macrophage membrane (Figure 6C, D). TEM showed that MM@CMANP had an additional membrane coating (red arrows), forming a core-shell structure, compared to CMANP (Figure 6E). Notably, the particle size observed by TEM represents the dry diameter, whereas DLS measures the hydrodynamic diameter in solution; therefore, DLS values are typically larger due to hydration and the soft membrane/protein layer. These data provide evidence for the successful integration of the macrophage membrane onto the CMANP surface. Additionally, SDS-PAGE and western blot analyses showed that the protein profile of MM@CMANP closely resembled that of the macrophage membrane (Figure S5F), and that CD11b, TNFR, and CCR2 were detectable on MM@CMANP (Figure 6F), indicating that the macrophage membrane antigens were preserved during the preparation process. The *in vitro* release kinetics of CMANP and MM@CMANP were subsequently tested. Even with the macrophage membrane coating, the release kinetics of MM@CMANP were similar to those of CMANP (Figure S5G).

### *In vitro* and *in vivo* inflammation targeting, biodistribution and mechanistic validation of macrophage membrane biomimetic nanoparticles

To evaluate whether macrophage membrane camouflage enhances inflammation targeting, rhodamine B-loaded PLGA nanoparticles (RBNP) or macrophage membrane-coated RBNP (MM@RBNP) were incubated with resting or LPS-stimulated RAW 264.7 macrophages. MM@RBNP exhibited markedly higher intracellular accumulation than uncoated RBNP in LPS-activated macrophages, indicating improved targeting efficiency toward inflamed macrophages (Figure 6G, H). Because inflammatory microenvironments are characterized by elevated chemokine gradients, leukocyte cell membrane-coated nanoparticles may inherit chemokine receptor-ligand interactions and migrate toward inflammatory lesions *via* the chemokine-chemokine receptor axis 37. Notably, CCL2-mediated recruitment has also been reported to promote the accumulation of macrophage membrane-camouflaged nanoparticles 38. Based on this rationale, we hypothesized that CCR2 retained on MM@RBNP contributes to its enhanced uptake in LPS-activated macrophages and therefore performed CCR2 knockdown-based validation. We established RAW 264.7 cells with stable CCR2 knockdown using two independent constructs (CCR2 KD1 and KD2), and knockdown efficiency was confirmed by western blotting (Figure S6A, B). Membranes derived from CCR2-knockdown macrophages were then used to coat RBNP, generating CCR2-deficient MM@RBNP. Compared with MM@RBNP prepared from native control membranes, CCR2-deficient MM@RBNP showed substantially reduced cellular accumulation in LPS-stimulated RAW 264.7 macrophages (Figure S6C, D). Collectively, these results demonstrate that macrophage membrane coating confers inflammation-targeting capability and that CCR2 on the membrane contributes significantly to MM@RBNP enrichment in activated macrophages, providing a mechanistic basis for the enhanced colonic accumulation observed *in vivo*.

For assessment of the targeting efficiency of macrophage membrane-coated nanoparticles to the site of colitis lesions, RBNP and MM@RBNP were administered in DSS-induced IBD mice *via* tail vein injection, and their accumulation in different organs was assessed. As shown in Figure 6H and 6I, MM@RBNP accumulation in the colon was 1.4 folds higher than that of RBNP, indicating that macrophage membrane-coated PLGA nanoparticles have enhanced targeting efficiency to colitis lesions. In addition, compared with the control group, the cumulative amounts of RBNP and MM@RBNP in the livers of DSS group mice were higher (Figure 6H, J), which may be due to DSS also induce inflammation in the liver 39. We further evaluated time-dependent biodistribution of RB-loaded nanoparticles in DSS-induced colitis mice. *Ex vivo* fluorescence imaging of major organs and colon collected at 1, 2, 4, 8 and 12 h displayed a steady elevation of fluorescence in the inflamed colon, reaching a maximum at ~8 h and remaining detectable at 12 h (Figure S7A). Quantitative analysis further indicated that colonic fluorescence of MM@RBNP was consistently higher than that of uncoated RBNP across the tested time points (Figure S7B), supporting that macrophage membrane coating enhances inflammation-associated enrichment. Organ distribution analysis at 8 h suggested that off-target signals were mainly observed in the liver and kidneys, while signals in the heart, lungs and spleen were minimal (Figure S7C).

In parallel, the pharmacokinetics of CMA delivered by MM@CMANP were assessed by measuring plasma CMA concentrations over time. The concentration-time profile displayed rapid systemic exposure followed by a slower elimination phase (Figure S7D). Non-compartmental analysis yielded a T_max_ of 0.05 h, C_max_ of 113.98 ± 32.1 ng/mL, and an apparent t_1/2_ of 5.72 ± 1.22 h, with an AUC0-∞ of 264.92 ± 41.73 ng·h/mL (Figure S7E). Notably, the apparent half-life of CMA after MM@CMANP administration was longer than the reported metabolic half-life of free CMA in rats (~1.7 h), suggesting that nanoformulation can prolong systemic exposure 40.

### MM@CMANP ameliorates colonic barrier damage in IBD mice

**Figure 7:**
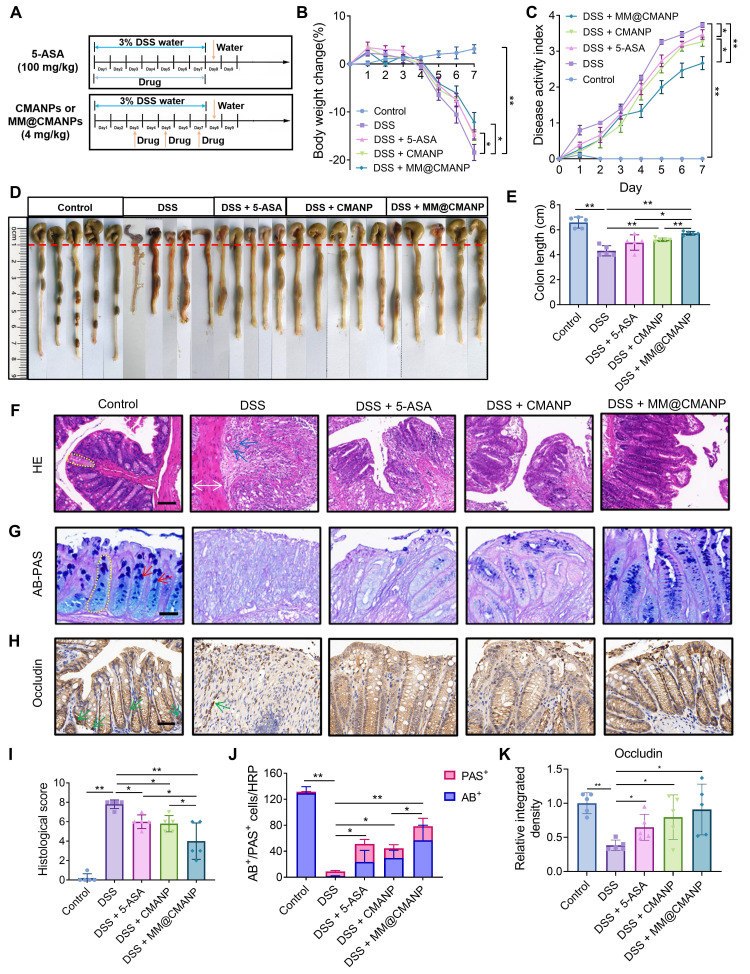
** MM@CMANP mitigates DSS-induced IBD in mice. (A)** Schematic diagram of the construction of a mouse IBD model and its treatment with 5-ASA or nanomedicines. **(B)** Weight change in IBD mice after treatment with 5-ASA, CMANP, or MM@CMANP (n = 5). **(C)** DAI of mice assessed by a composite score of the degree of weight loss, stool characters, and hematochezia (n = 5). **(D, E)** Comparison and statistical analysis of colon length from different treatment groups in IBD mice (n = 5). **(F, I)** Representative HE stained pathological sections and histological scores of IBD mice treated with different nanomedicines (n = 5). Scale bar: 100 μm. **(G, J)** Representative AB-PAS staining images and counts of acidic and neutral mucus produced by goblet cells in colonic tissue (n = 5). Scale bar: 50 μm. Yellow dashed outlines mark the crypt structures, white double-headed arrows indicate muscle layer thickness, blue arrows highlight inflammatory cell infiltration in H&E sections, red arrows indicate goblet cells in AB-PAS staining, and green arrows denote occludin-positive staining (brown) in the immunohistochemistry images. **(H, K)** Representative immunohistochemical staining images and relative quantitative analysis of occludin in colon tissues (n = 5). Scale bar: 50 μm. Data presented as mean ± SD, *P < 0.05, **P < 0.01.

For assessment of the therapeutic effect of CMA-loaded nanoparticles, a DSS-induced IBD mouse model was established (Figure 7A). Five-ASA was used as a positive control. After DSS treatment, the mice experienced a 18.5% weight loss by day 7. Treatment with 5-ASA, CMANP, or MM@CMANP significantly alleviated weight loss in IBD mice, and MM@CMANP exhibited a superior effect compared to the other groups (Figure 7B). Additionally, DSS-treated mice showed elevated DAI and shortened colon length. Similarly, MM@CMANP notably improved the DAI and colon length in IBD mice, outperforming both 5-ASA and CMANP (Figure 7C-E).

For evaluation of the pathological changes in the colonic tissue of the mice, HE staining was performed. DSS treatment resulted in disruption of the colonic mucosal barrier in mice, accompanied by loss of crypt structure, thickening of the mucosal muscle layer, and infiltration of inflammatory cells (Figure 7F). The treatment groups improved colonic barrier damage in IBD mice to varying degrees, with MM@CMANP showing the most pronounced effect (Figure 7I). Further evaluation of the changes in goblet cells in the colonic mucosal barrier was conducted through AB-PAS staining. AB/PAS double staining of control group sections demonstrated abundant goblet cells containing acidic (blue) and neutral (magenta) mucins 41. DSS treatment led to a marked reduction in goblet cells in the colonic mucosal barrier, whereas MM@CMANP significantly increased the number of goblet cells, with effects more pronounced than those of 5-ASA and CMANP (Figure 7G, J). Similarly, as an important tight junction protein, occludin had the highest expression level in the MM@CMANP-treated group compared to other treatment groups (Figure 7H, K). Overall, owing to its efficient delivery to IBD lesions in murine models, MM@CMANP exhibited superior therapeutic effects compared to the other treatment groups.

### MM@CMANP regulates macrophage polarization and promotes autophagy in IBD mice

**Figure 8:**
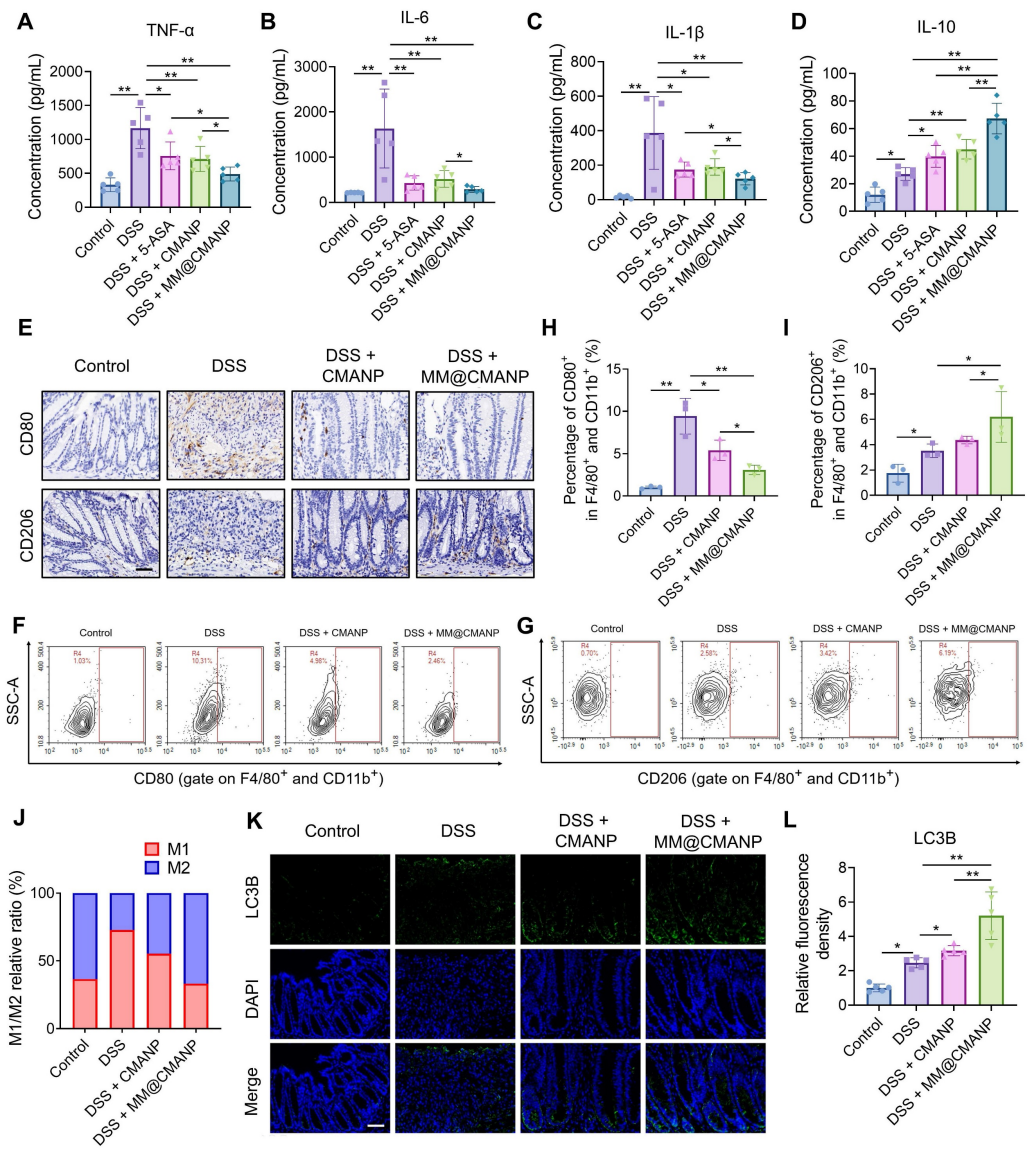
** MM@CMANP modulates macrophages polarization and enhances autophagy in IBD mice. (A-D)** Protein concentrations of TNF-α, IL-6, IL-1β and IL-10 in mouse serum were detected by ELISA assay (n = 5). **(E)** Representative immunohistochemical staining images of M1 marker CD80 and M2marker CD206 in mouse colon tissues. Scale bar: 50 μm. **(F, G)** Representative flow cytometry plots showing the frequencies of CD80^+^ (f) and CD206^+^ (g) cells gated on colonic lamina propria macrophages (F4/80^+^ and CD11b^+^) from Control, DSS, DSS + CMANP, and DSS + MM@CMANP mice. **(H, I)** Quantification of the percentages of CD80^+^ (h) and CD206^+^ (i) cells within F4/80^+^CD11b^+^ macrophages. **(J)** Relative M1/M2 ratio (or composition) calculated based on CD80^+^ and CD206^+^ macrophage populations (n = 3). **(K, L)** Representative immunofluorescence images and relative quantitative statistics of autophagosome marker LC3B in mouse colon tissues (n = 5). Scale bar: 50 μm. Data presented as mean ± SD, *P < 0.05, **P < 0.01.

To investigate whether CMA regulates macrophage polarization *in vivo*, we first measured serum levels of M1 macrophage-derived pro-inflammatory cytokines (TNF-α, IL-6, and IL-1β) and M2 macrophage-derived anti-inflammatory cytokine (IL-10) in mice. DSS administration triggered a marked elevation of pro-inflammatory cytokines (TNF-α, IL-6, IL-1β) as well as IL-10 in the serum, contrasting sharply with control values. Treatment with 5-ASA, CMANP, or MM@CMANP significantly reduced TNF-α, IL-6, and IL-1β levels (Figure 8A-C) while increasing IL-10 levels (Figure 8D), with MM@CMANP demonstrating superior effects compared to 5-ASA and CMANP. Immunohistochemistry was then performed to assess CD80 and CD206 expression in mouse colonic tissues. DSS-treated mice displayed elevated levels of both CD80 and CD206 compared to the control group, indicating the activation of both M1 and M2 macrophages during IBD progression, consistent with previous reports 42. CMANP and MM@CMANP treatment suppressed CD80 expression while promoting CD206 expression (Figure 8E). Flow cytometry of colonic lamina propria cells showed that DSS markedly increased the proportion of CD80⁺ (M1-like) macrophages and decreased CD206⁺ (M2-like) macrophages within the F4/80⁺CD11b⁺ population, whereas MM@CMANP significantly reversed this polarization shift. Accordingly, MM@CMANP reduced the M1/M2 ratio compared with DSS and CMANP groups, in line with the immunohistochemistry observations (Figure 8F-J). These findings suggest that CMA-loaded nanoparticles mitigated M1 polarization and enhanced M2 polarization in IBD mouse colons. Owing to its targeted delivery, MM@CMANP displayed superior performance in this regard.

To investigate whether CMA-loaded nanoparticles enhance autophagy *in vivo*, we performed immunofluorescence staining of LC3B in mouse colonic tissues. DSS treatment increased LC3B expression in the colonic tissues compared to the control group, and treatment with CMANP or MM@CMANP further enhanced LC3B expression (Figure 8I, J), indicating that CMA promotes autophagy in IBD mice. In summary, consistent with *in vitro* findings, these observations reveal that MM@CMANP exerts protective effects in IBD mice by modulating macrophage polarization and enhancing autophagy.

## Discussion and Conclusion

As a key component of the innate immune system, macrophage functions as a pivotal regulator in the pathogenesis of IBD. Rebalancing M1/M2 macrophages offers a novel therapeutic avenue for inducing IBD remission 6, 43. Certain anti-inflammatory agents, such as bardoxolone methyl and the IKK inhibitor TPCA-1, demonstrate dual regulatory effects by suppressing macrophage M1 and M2 polarization 44. This concurrent inhibition may weaken their therapeutic efficacy in inflammation-associated diseases. Thus, identifying compounds that selectively inhibit M1 polarization while maintaining M2 functionality represent a valuable therapeutic strategy for IBD. In this study, we report that CMA orchestrates a phenotypic switch in macrophages, attenuating M1 and augmenting M2 polarization, thereby supporting its therapeutic utility in IBD.

Distinct metabolic profiles of M1 and M2 macrophages critically influence their polarization and function. IFN-γ/LPS-activated M1 macrophages exhibit a metabolic shift toward aerobic glycolysis, which supports rapid energy demands for pro-inflammatory and antimicrobial responses 31. In contrast, IL-4/IL-13-activated M2 macrophages primarily rely on oxidative phosphorylation to meet energy requirements for synthesizing anti-inflammatory mediators and tissue remodeling 7. While metabolic reprogramming during macrophage polarization was initially viewed as a passive adaptation to energy demands, emerging evidence suggests that modulating these metabolic pathways can actively reprogram macrophage phenotypes. A glycolytic bias drives M1 polarization, and pharmacological inhibition of glycolysis reverses this process 45. Conversely, enhancing oxidative phosphorylation promotes M2 polarization 46. Our results demonstrate that CMA suppresses the glycolysis in M1 macrophages while partially restoring oxidative phosphorylation. Notably, CMA also restricts glucose uptake by downregulating GLUT1. These observations align with the concept that metabolic reprogramming serves as a regulatory checkpoint for macrophage polarization.

Mitochondria, as central hubs regulating metabolic and inflammatory responses, are important for maintaining macrophage polarization. In M1 macrophages, enhanced aerobic glycolysis shifts mitochondrial function from ATP synthesis to mtROS production 47. Excessive mtROS induce mitochondrial dysfunction and activate the NLRP3 inflammasome, further exacerbating the pro-inflammatory M1 state 33. As a mitochondrial quality-control mechanism, mitophagy selectively clears dysfunctional mitochondria, while impaired mitophagy leads to dysfunctional mitochondrial accumulation, promoting the progression of inflammation 35, 48. Here, CMA reduced mtROS levels, transiently enhanced lysosomal activity and mitophagy flux, and accelerated the removal of dysfunctional mitochondria. Mechanistically, CMA-induced mitophagy in M1 macrophages was mediated *via* the BNIP3 pathway, independent of the canonical PINK1-Parkin pathway. The reversal of CMA's anti-M1 effects by autophagy inhibitors (CQ and Baf A1) and BNIP3 knockdown further confirms the centrality of mitophagy in its mechanism.

In view of the suboptimal efficacy and adverse effects associated with current IBD therapies, developing safe and targeted delivery systems is imperative. In this study, the engineering of a macrophage membrane-coated nano-delivery system provides an effective means to surmount the pharmacokinetic limitations inherent to CMA. MM@CMANP achieves targeted delivery to inflamed colonic tissues by leveraging the inherent inflammation-homing capability of macrophage. In the current work, MM@CMANP was administered intravenously as a proof-of-concept to ensure reliable systemic exposure and to minimize variability associated with gastrointestinal transit and absorption. Compared with oral administration, intravenous injection bypasses potential barriers in the gastrointestinal tract, thereby facilitating a clearer evaluation of membrane-mediated targeting and colonic enrichment. Nevertheless, intravenous administration may be less practical for long-term use. Therefore, future studies will explore optimization of dosing regimens and alternative administration routes, together with comprehensive safety assessment under repeated dosing. *In vivo*, MM@CMANP outperformed both CMANP and 5-ASA, as evidenced by restored colon length, reduced disease activity index, and improved mucosal integrity. Accumulating research shows that M1 macrophage polarization drives the pathological progression of IBD. Clinically approved therapeutic drugs such as infliximab and adalimumab target TNF-α produced by M1 macrophages to mitigate IBD symptoms 49. In contrast, M2 macrophages mediate tissue repair, contributing to the restoration of the mucosal barrier. Elevated M2 macrophage infiltration in the murine colon enhances resistance to DSS-induced IBD 50, while administration of *in vitro*-generated M2 macrophages into mice attenuates colitis 51. Furthermore, knockdown of the M2-associated cytokine IL-10 exacerbates colitis development in murine models 52. In this study, MM@CMANP suppressed M1 polarization, enhanced M2 polarization, and upregulated autophagy in IBD mice, indicating that CMA exerts analogous biological mechanisms *in vivo*. Despite the promising therapeutic outcomes of MM@CMANP, several limitations should be acknowledged. Although CMA-loaded PLGA nanoparticles (CMANP) were included as a formulation-matched control to isolate the incremental benefit of macrophage membrane camouflage, we did not evaluate a free (CMA-only) treatment group *in vivo*. Future studies will incorporate a rigorously validated free-CMA formulation to enable a more direct comparison and to further quantify the added value of nanoparticulate delivery.

In summary, this study establishes CMA as a regulator of macrophage polarization through BNIP3-mediated mitophagy, and demonstrates the therapeutic promise of macrophage membrane biomimetic nano-delivery system in IBD. Our findings provide a preclinical foundation for CMA-based therapies and introduce MM@CMANP as a novel strategy to enhance drug targeting and efficacy, offering new avenues for managing chronic inflammatory diseases.

## Supplementary Material

Supplementary methods, figures and table.
