# Supplementary material for: Macrophage membrane-biomimetic cinnamaldehyde nanomedicine ameliorates inflammatory bowel disease by suppressing macrophage M1 polarization
Source: Theranostics. 2026 Mar 25;16(10):5589–608. doi: 10.7150/thno.124748 (PMC13080821; doi:10.7150/thno.124748)
Supplement: Supplementary file 1 — Supplementary methods, figures and table. [file thnov16p5589s1.pdf]

Supporting Information

Methods

Cell viability test

The effect of cinnamaldehyde (CMA) on the viability of RAW 264.7 macrophages was assessed using the MTT assay. Briefly, RAW 264.7 cells (5×10^3 cells per well) were seeded into a 96-well plate and allowed to adhere for 24 hours. Subsequently, the cells were exposed to CMA at concentrations of 0, 5, 10, 20, 40, and 80 μg/mL for an additional 24 hours. Following incubation, the medium was discarded, and 100 μL of MTT solution (0.5 mg/mL) was added and incubated for 4 hours. The supernatant was then removed, and the resulting formazan crystals were dissolved in 100 μL of DMSO per well. Absorbance was measured at 570 nm, and the results were expressed as percentages.

Detection of intracellular and mitochondrial ROS

Intracellular ROS and mitochondrial ROS levels in the RAW 264.7 macrophages were measured using the DCFH-DA and MitoSOX Red fluorescent probe, respectively. RAW 264.7 cells were plated in a 6-well plate at a density of 1×10^6 cells per well and cultured for 24 hours. The cells were then treated with CMA (20 μM) for 1 hour, followed by co-treatment with LPS (1 μg/mL) for an additional 24 hours. For the detection of intracellular ROS, DCFH-DA was added for 30 minutes, and the cell samples were then imaged using the EVOS FL Auto Cell Imaging System (ThermoFisher, Waltham, MA, USA), with an excitation wavelength of 488 nm and an emission wavelength of 525 nm. For the detection of mitochondrial ROS, MitoSOX Red was added for 30 minutes. The cell samples were then analyzed using flow cytometry (BD, New Jersey, USA) and imaged using the EVOS FL Auto Cell Imaging System, with an excitation wavelength of 396 nm and an emission wavelength of 610 nm.

RNA sequencing analysis

For RNA sequencing analysis, three groups (each with three biological replicates) were included: control, LPS, and LPS + CMA. Total RNA was extracted from each

group using Trizon reagent. After verifying RNA purity, concentration, and integrity to ensure sample quality, cDNA libraries were prepared and sequenced on the DNBSEQ-T7 with PE150 model at Wuhan GeneCreate Biological Engineering Co., Ltd.

Detection of autophagosomes

Autophagosomes were detected using the Monodansyl cadaverine (MDC) fluorescent probe. Briefly, RAW 264.7 cells were seeded at a density of 5×10^5 cells/mL into 35 mm confocal culture dishes. After 24 hours, the cells were pretreated with CMA (20 μM) for 1 hour, followed by co-treatment with LPS (1 μg/mL). Autophagy was assessed at various time points (0, 2, 4, 8, 16, and 24 hours) after LPS treatment. Cells were incubated with MDC (10 μM) for 30 minutes and then imaged under a laser scanning confocal microscope. The excitation and emission wavelengths for MDC were 405 nm and 450 nm, respectively. The total fluorescence intensity of autophagosomes in each cell was quantified using ImageJ.

Preparation of CMA-loaded PLGA nanoparticles (CMANP)

CMANP were prepared *via* the nanoprecipitation method. Briefly, 80 mg of PLGA was dissolved in 4 mL of acetone, followed by the addition of 20 mg of CMA. The mixture was vortexed thoroughly to ensure homogeneity. The organic phase was then added dropwise into 10 mL of deionized water under continuous stirring at 600 rpm for 2 h at room temperature. The resulting suspension was centrifuged at 2000 rpm for 5 min to remove aggregated PLGA and large particulates. The supernatant was collected and dialyzed against deionized water using a dialysis membrane (MWCO: 3500 Da) for 24 h to eliminate unencapsulated CMA and residual acetone. The purified CMANP dispersion was stored at 4°C for further use. Rhodamine B-loaded nanoparticles (RBNP) were synthesized following an identical protocol, with 0.1 wt% rhodamine B (RB) substituted for CMA.

Characterization of the nanoparticles

The particle size, polydispersity index (PDI) and zeta potential of the nanomedicines were analyzed by dynamic light scattering (DLS) using a laser diffraction particle size analyzer (Mastersizer 3000; Malvern Panalytical, UK). For particle size analysis, the sample was loaded into a cuvette, and measurements were

performed using the integrated "BIC Particle Sizing" software under automatic optical alignment. For zeta potential determination, the AQ-748 electrode was mounted into a cuvette. The sample was injected into the cuvette, and measurements were conducted using the "BIC Zeta Potential Analyzer" software.

Analysis of encapsulation efficiency and loading efficiency of CMA

CMA was diluted to 0.125, 0.25, 0.5, 1, 2 and 4 μg/mL with DMSO to establish a standard curve. Subsequently, CMANP were lyophilized and dissolved in DMSO, and the absorbance was measured using a UV-Vis spectrophotometer at 289 nm. Based on the standard curve, the encapsulation efficiency (EE) and loading efficiency (LE) of CMA were calculated as follows:

$$EE\ (\%) = \frac{M_{CMA}}{M_{added}} \times 100\%$$

$$LE\ (\%) = \frac{M_{CMA}}{M_{CMA} + M_{PLGA}} \times 100\%$$

where M_{CMA} is the mass of CMA loaded in the nanoparticles, M_{PLGA} is the mass of PLGA in the formulation and M_{added} is the mass of CMA added.

Stability evaluation of CMANP or MM@CMANP

The stability of CMANP stored at 4°C was evaluated at predetermined time points (days 1, 4, 9, 14, 21, and 28 post-preparation). Briefly, samples were retrieved from storage and equilibrated to room temperature for 30 min. Nanoparticle size and zeta potential were analyzed by using laser diffraction particle size analyzer (Mastersizer 3000). The colloidal stability of MM@CMANP under physiological conditions was evaluated by incubating the nanoparticles in mouse serum at 37 °C for 3 days, during which the hydrodynamic diameter was measured at the indicated time points by DLS.

Lentiviral shRNA–mediated stable CCR2 knockdown

Stable CCR2-knockdown RAW264.7 macrophages were generated using lentiviral shRNA. Two shRNAs targeting mouse Ccr2 were designed and cloned into a lentiviral shRNA vector (CCR2 KD1 and CCR2 KD2). The target sequences were: shRNA1, 5’-CCTCTCTACCAGGAATCATAT-3’ and shRNA2, 5’-GCAACATGTTGGTCATTATAA-3’. A non-targeting shRNA was used as a negative control. Lentiviruses were produced in HEK293T cells by co-transfecting the shRNA

plasmids with packaging plasmids. Viral supernatants were harvested at 48–72 h post-transfection, clarified by centrifugation, and filtered (0.45 μm). RAW264.7 cells were infected with the lentiviral particles, followed by antibiotic selection to obtain stable cell populations. CCR2 knockdown efficiency was verified by western blotting prior to subsequent experiments, including membrane extraction and nanoparticle coating.

Pharmacokinetic study and plasma sample preparation

A staggered sparse-sampling design was used to obtain a composite plasma concentration – time profile in mice. Six male C57BL/6 mice (~8 weeks old) were randomly assigned to two groups (Group A and Group B; n = 3 per group). Following tail-vein injection of MM@CMANP (100 μL per mouse), blood samples (~50 μL each) were collected from the tail vein at four time points per group: Group A at 3, 8, 15, and 30 min, and Group B at 1, 2, 4, and 8 h. Blood samples were immediately centrifuged at 3,000 rpm for 10 min at 4°C, and the plasma supernatant was transferred to clean microcentrifuge tubes for analysis. Plasma concentrations of CMA were quantified using a triple quadrupole LC–MS/MS system, with α-methylcinnamaldehyde as the internal standard.

Supplementary Table

Table S1. Primer sequences of M1 and M2 markers

Gene	Name	Primer sequences (5’-3’)
Nos2	Forward primer	AACGGAGAACGTTGGATTTG
	Reverse primer	CAGCACAAGGGGTTTTCTTC
Cd80	Forward primer	ACCCCCAACATAACTGAGTCT
	Reverse primer	TTCCAACCAAGAGAAGCGAGG
Tnf	Forward primer	CCTCACCCACACCGTCAG
	Reverse primer	GTTGGTCCCCCTTCTCCA
Arg1	Forward primer	GCTGTCTTCCCAAGAGTTGGG
	Reverse primer	ATGGAAGAGACCTTCAGCTAC
Cd206	Forward primer	CTCTGTTCAGCTATTGGACGC
	Reverse primer	CGGAATTTCTGGGATTCAGCTT
Actb	Forward primer	GTGACGTTGACATCCGTAAAGA
	Reverse primer	GTAACAGTCCGCCTAGAAGCAC

Supplementary Figure

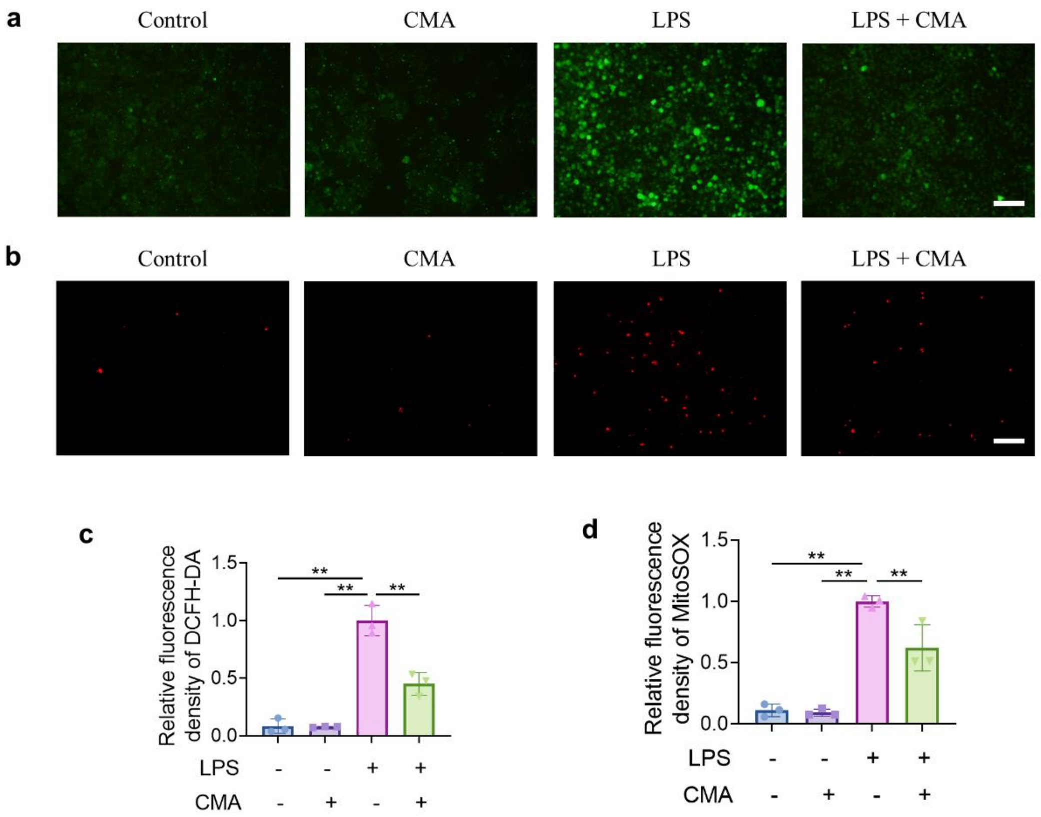

Figure S1. CMA inhibits intracellular and mitochondrial-derived ROS of M1 macrophages. RAW 264.7 macrophages were pretreated with CMA (20 μM) for 1 h, followed by co-treatment with LPS (1 μg/mL) for 24 h. (**A, C**) Representative intracellular ROS images and relative quantitation of RAW 264.7 macrophages detected by DCFH-DA (n = 3). (**B, D**) Representative mitochondrial-derived ROS images and relative quantitation of RAW 264.7 macrophages detected by MitoSOX (n = 3). Scale bar: 100 μm. Data are expressed as mean ± SD, $^*P < 0.05$, $^{**}P < 0.01$.

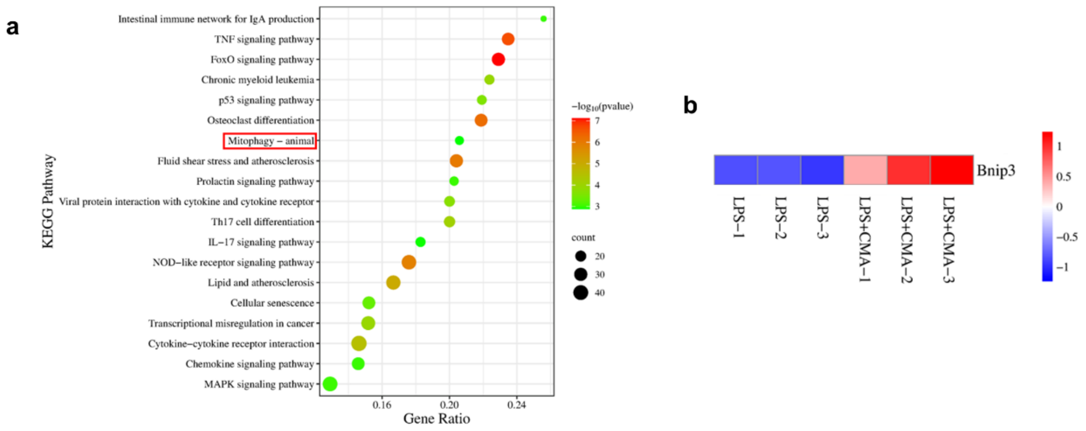

Figure S2. RNA sequencing of LPS-induced RAW 264.7 macrophages with or without CMA (20 μM) treatment. (**A**) KEGG enrichment analysis of DEGs in the LPS group vs. LPS + CMA group. (**B**) Relative FPKM values of *Bnip3* gene in the LPS group vs. LPS + CMA group.

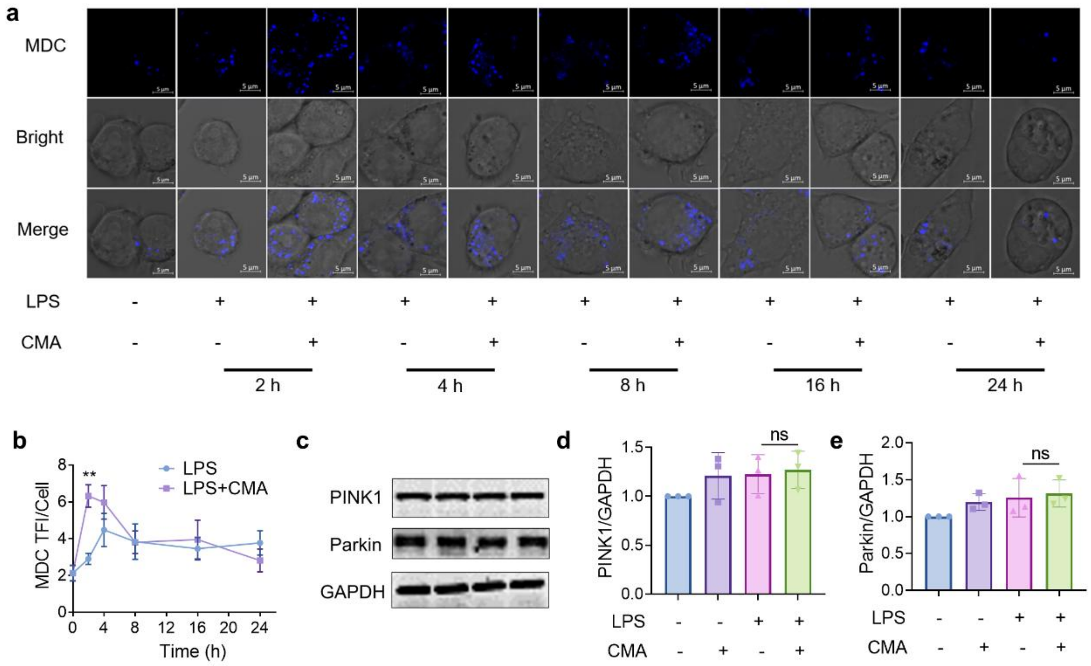

Figure S3. Effect of CMA on mitophagy in M1 macrophages. (**A, B**) Representative images and quantitative analysis of MDC-labeled autophagosomes in M1 macrophages with or without CMA (20 μM) treatment. Quantitative analysis involved counting MDC fluorescence in each cell within four to eight randomly selected fields. Scale bar: 5 μm. (**C-E**) Representative immunoblot bands and quantification of PINK1 and Parkin in M1 macrophages treated with or without CMA for 2 h (n = 3). Data are expressed as mean ± SD, **P < 0.01, ns, not significant.

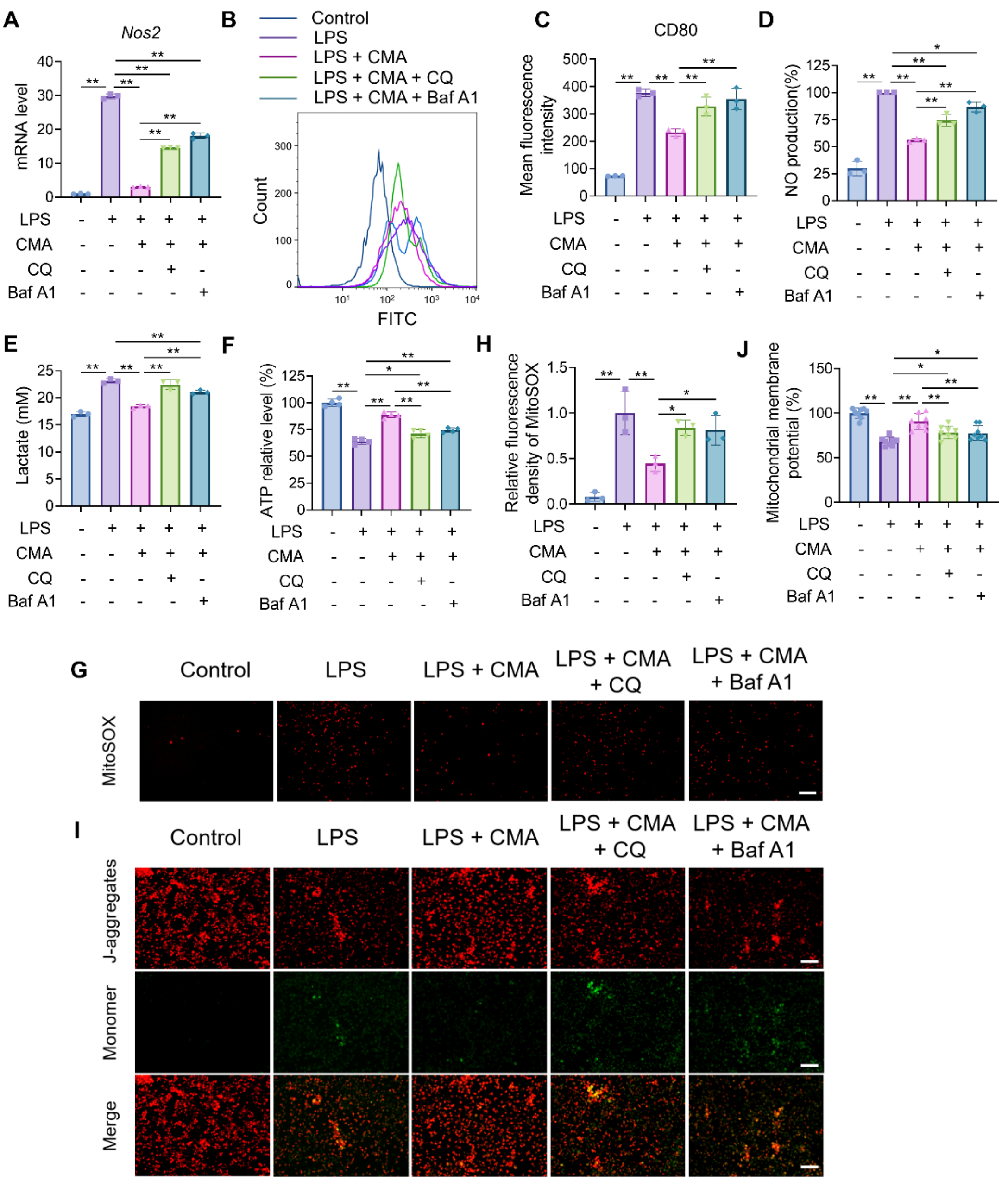

Figure S4. Pharmacological blockade of reverses the suppressive effect of CMA on M1 markers and mitochondrial dysfunction. RAW 264.7 macrophages were pretreated with CMA (20 μM) for 1 h, followed by co-treatment with LPS (1 μg/mL) for 24 h. (**A**) The mRNA level of *Nos2* in RAW 264.7 macrophages (n = 3). (**B, C**) Flow cytometry analysis and quantification of CD80 protein levels in RAW 264.7 macrophages (n = 3). (**D, E**) NO levels and lactate production in the supernatants of RAW 264.7 macrophages (n = 3). (**F**) Intracellular ATP levels of RAW 264.7 macrophages (n = 4). (**G, H**) Representative fluorescence images and quantitative analysis of mtROS in RAW 264.7 macrophages (n = 3). Scale bar: 100 μm. (**I, J**) Fluorescence microscopy and fluorescence microplate reader analysis of JC-1-labeled mitochondrial membrane potential in RAW 264.7 macrophages. Data are expressed as mean ± SD, $*P < 0.05$, $**P < 0.01$.

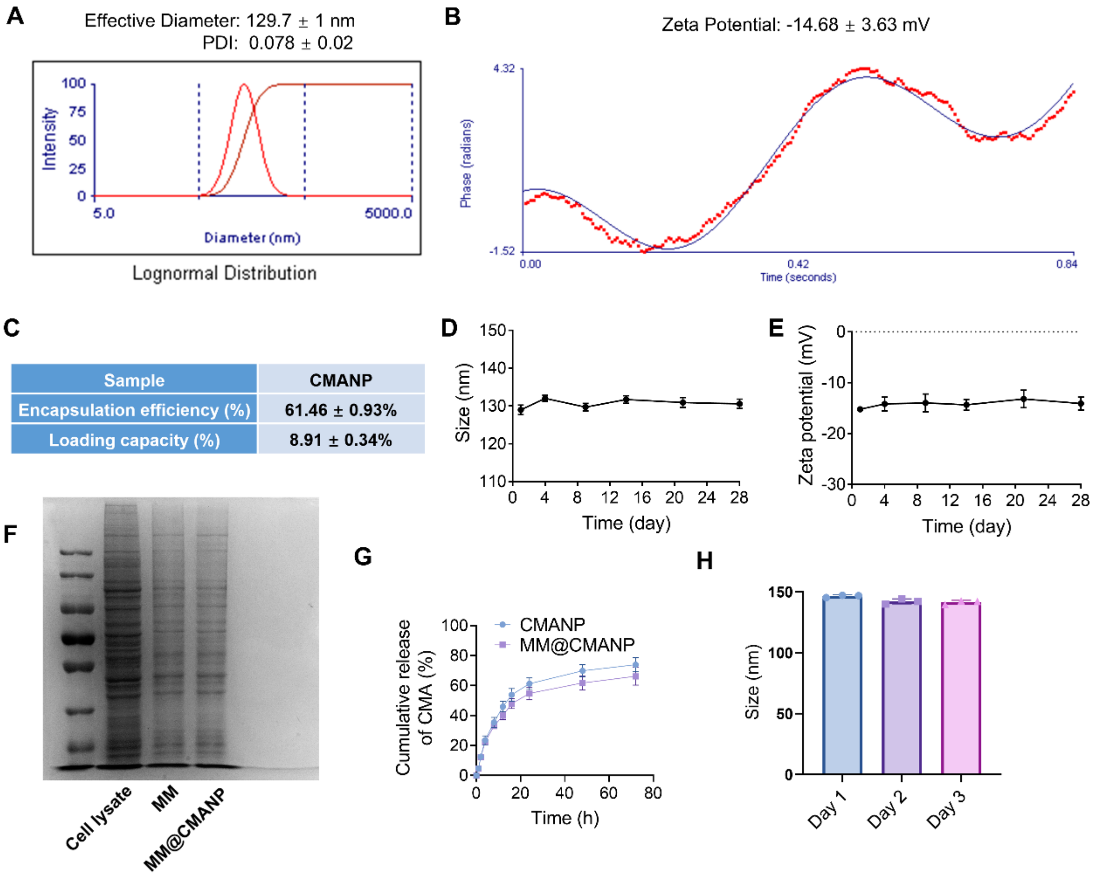

Figure S5. Preparation and characterization of CMANP. (**A, B**) The effective diameter, PDI and zeta potential of CMANP analyzed by DLS. (**C**) The encapsulation efficacy and loading capacity of CMA in PLGA nanoparticles. (**D, E**) Changes in size and zeta potential of CMANP stored in 4°C water for 28 days. (**F**) Protein profiles of RAW 264.7 cell lysate, MM and MM@CMANP detected by SDS-PAGE. (**G**) Release curves of CMANP and MM@CMANP *in vitro*. (**H**) Stability testing of MM@CMANP in mouse serum. Data are expressed as mean ± SD (n = 3).

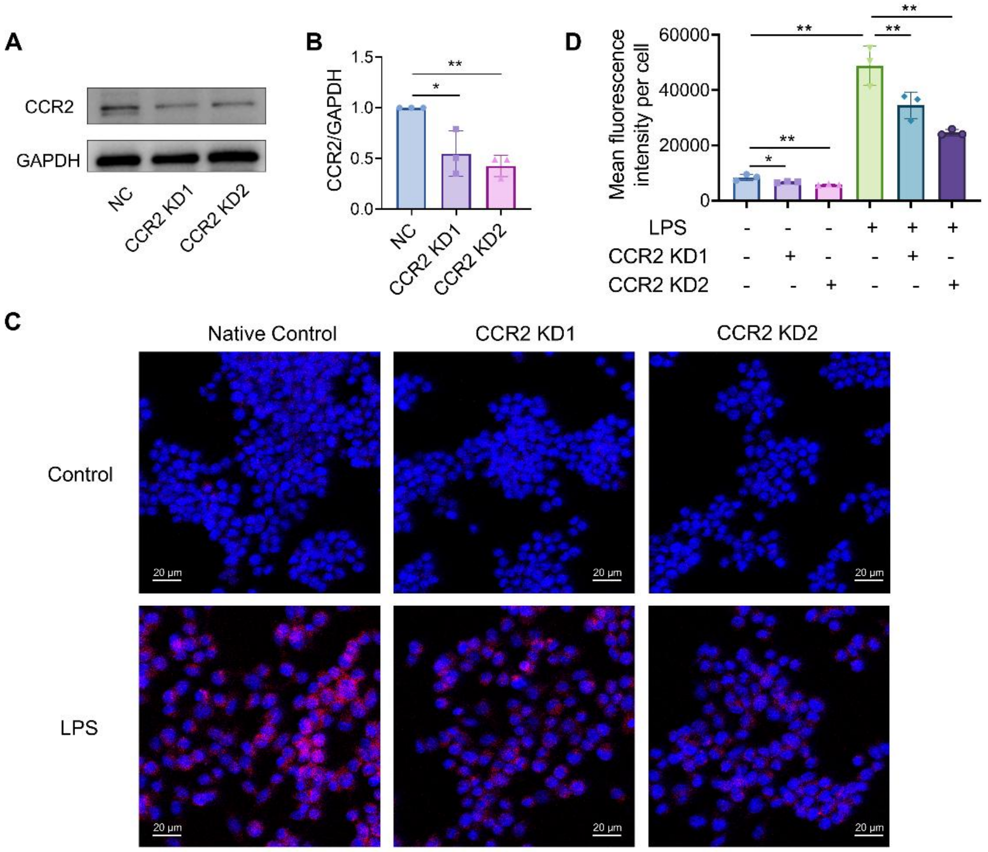

Figure S6. Establishment of CCR2-knockdown RAW264.7 macrophages and CCR2-dependent cellular uptake of MM@RBNP. (**A**) Western blot analysis of CCR2 expression in native control (NC) and CCR2 knockdown RAW264.7 cells (CCR2 KD1 and CCR2 KD2). (**B**) Quantification of CCR2 normalized to GAPDH. (**C**) Representative fluorescence images showing cellular accumulation of MM@RBNP prepared from NC or CCR2-knockdown membranes in resting (Control) or LPS-stimulated RAW264.7 cells; nuclei were counterstained with DAPI (blue) and nanoparticles are shown in red. Scale bar: 20 μm. (**D**) Quantification of intracellular fluorescence (mean fluorescence intensity per cell) corresponding to panel (C). Data are presented as mean ± SD (n = 3), *$P < 0.05$, **$P < 0.01$.

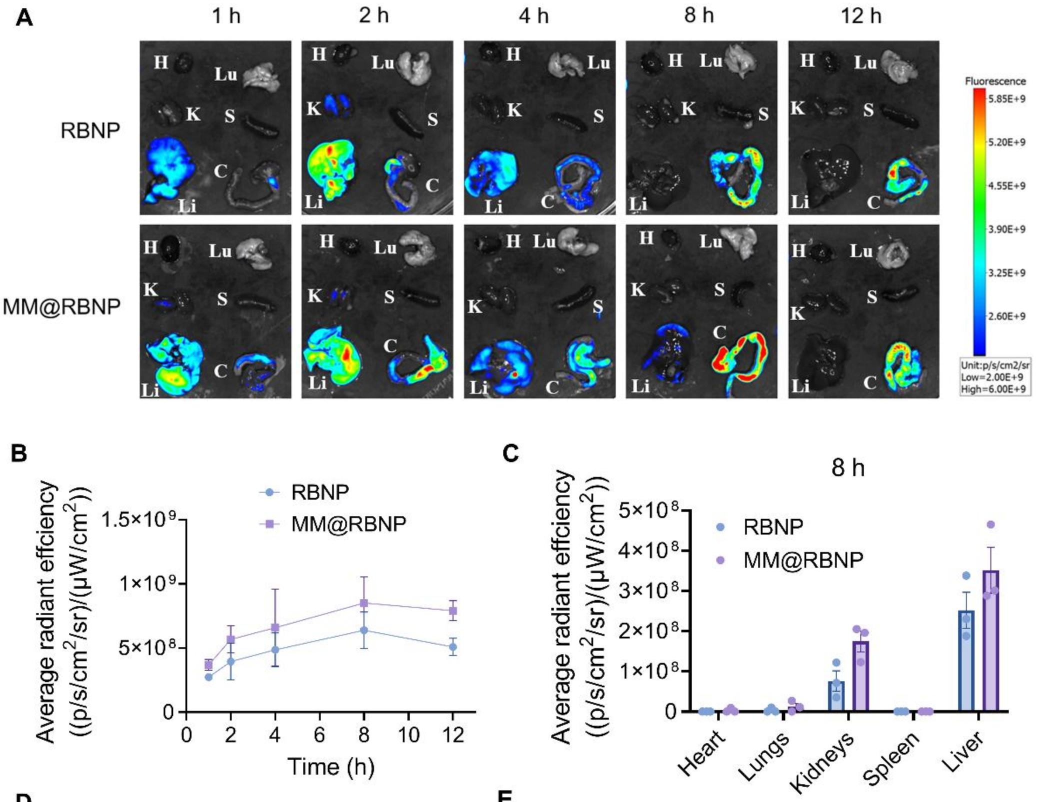

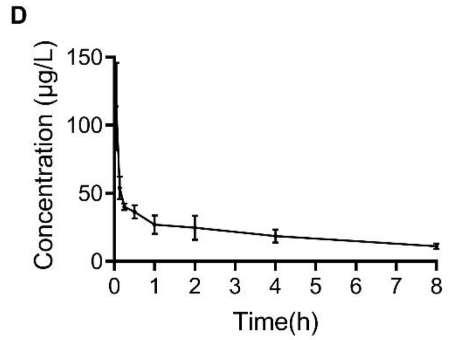

E

Parameter	Cinnamaldehyde
AUC_{0-t} (ng·h/mL)	172.35 ± 31.82
$AUC_{0-\infty}$ (ng·h/mL)	264.92 ± 41.73
MRT (h)	1.01 ± 0.0042
$t_{1/2}$ (h)	5.72 ± 1.22
T_{max} (h)	0.05
CL_z/F (L/h/kg)	15.37 ± 2.63
V_z/F (L/kg)	127.01 ± 34.83
C_{max} (ng/mL)	113.98 ± 32.1

Figure S7. Time-dependent biodistribution of macrophage membrane-coated nanoparticles in DSS-induced colitis mice and pharmacokinetics of MM@CMANP. (**A**) Representative *ex vivo* fluorescence images of major organs and colon harvested at 1, 2, 4, 8, and 12 h after intravenous administration of RBNP or MM@RBNP in DSS-induced colitis mice. (**B**) Quantification of fluorescence intensity in the colon at the indicated time points. (**C**) Organ distribution analysis of fluorescence intensity at 8 h post-injection. H, Lu, K, S, Li, and C represent the heart, lung, kidney, spleen, liver, and colon, respectively. (**D**) Plasma concentration-time profile of CMA after intravenous administration of MM@CMANP in mice. (**E**) Pharmacokinetic parameters of CMA following MM@CMANP administration calculated by non-compartmental analysis. Data are presented as mean ± SD (n = 3).
